# Combined Electrochemical, XPS, and STXM Study of Lithium Nitride as a Protective Coating for Lithium Metal and Lithium–Sulfur Batteries

**DOI:** 10.1021/acsami.3c04897

**Published:** 2023-08-08

**Authors:** Samuel D. S. Fitch, Gilles E. Moehl, Nina Meddings, Sacha Fop, Samantha Soulé, Tien-Lin Lee, Majid Kazemian, Nuria Garcia-Araez, Andrew L. Hector

**Affiliations:** † School of Chemistry, University of Southampton, Southampton SO17 1BJ, U.K.; ‡ Diamond House, Harwell Science and Innovation Campus, Diamond Light Source Ltd, Didcot OX11 0DE, Oxfordshire, U.K.

**Keywords:** lithium metal anode, protective coating, artificial SEI, lithium–sulfur batteries, polysulfides, XPS, STXM, operando optical microscopy

## Abstract

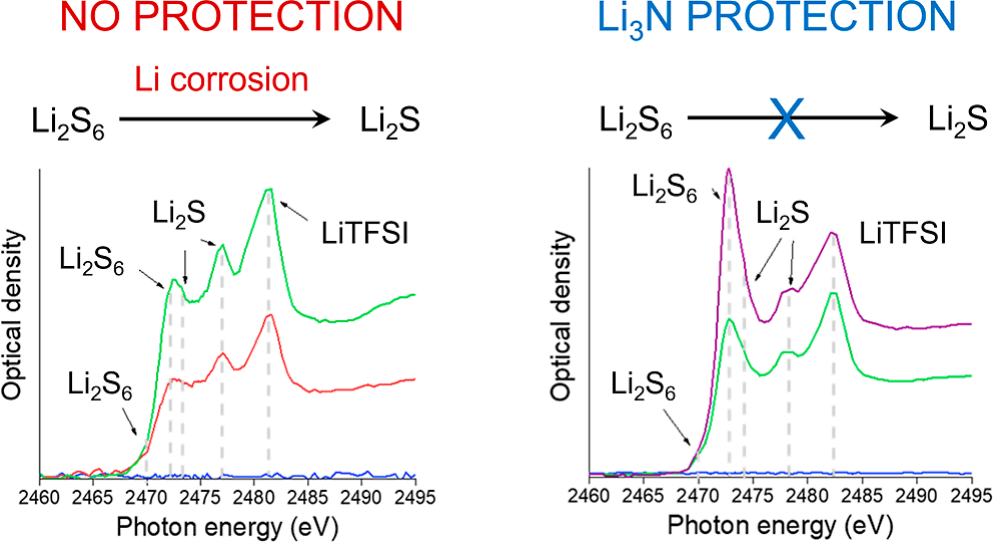

Li_3_N is an excellent protective coating material for lithium electrodes with very high lithium-ion conductivity and low electronic conductivity, but the formation of stable and homogeneous coatings is technically very difficult. Here, we show that protective Li_3_N coatings can be simply formed by the direct reaction of electrodeposited lithium electrodes with N_2_ gas, whereas using battery-grade lithium foil is problematic due to the presence of a native passivation layer that hampers that reaction. The protective Li_3_N coating is effective at preventing lithium dendrite formation, as found from unidirectional plating and plating–stripping measurements in Li–Li cells. The Li_3_N coating also efficiently suppresses the parasitic reactions of polysulfides and other electrolyte species with the lithium electrode, as demonstrated by scanning transmission X-ray microscopy, X-ray photoelectron spectroscopy, and optical microscopy. The protection of the lithium electrode against corrosion by polysulfides and other electrolyte species, as well as the promotion of smooth deposits without dendrites, makes the Li_3_N coating highly promising for applications in lithium metal batteries, such as lithium–sulfur batteries. The present findings show that the formation of Li_3_N can be achieved with lithium electrodes covered by a secondary electrolyte interface layer, which proves that the in situ formation of Li_3_N coatings inside the batteries is attainable.

## 1 Introduction

Rechargeable lithium metal batteries were first reported in the early 1960s, when W.S. Harris found that Li metal could be electrochemically plated in organic liquid electrolytes, albeit with low Coulombic efficiency.^1^ However, growing safety concerns due to short-circuit accidents induced by dendritic growth, combined with poor cyclability, shifted the research focus to lithium-ion batteries, and Sony commercialized a battery that replaced the lithium metal with a carbonaceous anode in 1991.^2^ More recently, the popularization of electric vehicles has increased the demand for high energy batteries, which has brought lithium metal-based batteries back into the spotlight. The issues of dendritic growth and poor cyclability still hamper the commercialization of lithium metal batteries, but the advancement of characterization techniques is a unique asset to support their development.

Lithium–sulfur batteries are a particularly promising type of lithium metal battery, due to low cost and high abundance of sulfur, but the formation of soluble lithium polysulfides triggers parasitic reactions with the lithium metal anode that severely compromise performance.^3^ Like for any other lithium metal battery, the development of effective lithium protection approaches is crucial to suppressing the issue of accelerated electrolyte degradation that leads to poor cycling and uneven lithium deposition. In addition, for lithium–sulfur batteries, the lithium protection also plays a key role in suppressing the parasitic reaction of polysulfides with the lithium electrode.

The formation of a protective lithium nitride (Li_3_N) coating from the reaction of lithium metal with nitrogen has been proposed as an effective approach to suppress the instability of lithium in battery electrolytes.^4^ Li_3_N is highly advantageous because it has an outstanding lithium conductivity and very low electronic conductivity.^5^ However, the formation of stable and uniform Li_3_N coatings on lithium metal is technically very challenging.

Aurbach et al. used mixtures of ball-milled Li_3_N and lithium metal as the anode of AA cells with a Li_0.3_MnO_2_ cathode and an electrolyte made of 1 M LiAsF_6_ in 1,3-dioxolane (DOL) with 1000 ppm of tributylamine (TBA). They found that the cycle life was inferior to that of batteries with a pure lithium metal anode, despite the fact that the presence of Li_3_N was found to decrease lithium metal corrosion.^6^ Wu et al. found that exposing lithium metal anodes to a N_2_ atmosphere for a limited time (1 h) produced improvements in the plating/stripping efficiency of cells with a polished copper working electrode, a lithium metal counter and reference electrode (with or without the Li_3_N formed upon N_2_ exposure), and a 1 M LiPF_6_ in the ethylene carbonate/dimethyl carbonate electrolyte.^7^ Zhang et al. further expanded on these conditions by varying the N_2_ gas flow rate and reaction temperature and found that the longest cycle life was obtained when the lithium metal electrodes were reacted with N_2_ at 20 °C.^8^ A similar approach was taken by Ma et al., who produced Li_3_N-protected lithium metal anodes for use in Li–S batteries, from the controlled reaction of lithium metal with N_2_, obtaining significant improvements in cycle life for Li–S cells containing a 1 M lithium bis(tri fluoromethanesulfonyl)imide (LiTFSI) in the 1,3-dioxolane/dimethyl ether electrolyte.^9^

Later on, Armand and co-workers studied five different approaches for the production of Li_3_N coatings on lithium for Li–S batteries, including an in situ generation method in which azido-trimethyl silane ((CH_3_)_3_SiN_3_), added to the electrolyte as an additive or directly drop coated onto lithium, was used to form the Li_3_N coating upon decomposition in contact with the lithium electrode.^10^ Park and Goodenough coated a bed of Li_3_N nanoparticles on lithium electrodes, which provided stable lithium plating/stripping without dendrite formation even at high current densities of >1 mA cm^–2^ with high capacities of 6 mA h cm^–2^.^11^ More recently, Cui and co-workers developed a method to produce a robust, pinhole-free Li_3_N coating on lithium from the reaction of freshly formed lithium surfaces (obtained by rapidly pulling apart a Cu/Li/Cu sandwich at 450 °C) with N_2_, with further heating for 1 s at 450 °C.^12^ The pinhole-free Li_3_N coating effectively prevented dendrite growth and enhanced the cycling life of all-solid-state Li|pinholefree-Li_3_N|Li symmetric cells and of cells with a Li_4_Ti_5_O_12_ counter electrode and 1 M LiTFSI in DOL/DME with 1 wt % LiNO_3_ liquid electrolyte.

The previous studies clearly show the promise of using a Li_3_N coating to protect metallic lithium to improve its electrochemical performance and suppress dendrite formation, but the development of a suitable procedure to form such a coating still needs further investigation. The use of molten lithium^4,6,12^ or the highly hygroscopic Li_3_N^4,6,10,11^ brings safety concerns for commercial applications, whereas the direct reaction of lithium metal with N_2_(g)^7–9^ is much simpler. However, this work shows that the latter reaction is intrinsically irreproducible when applied to lithium foils due to the presence of a native oxide layer on lithium electrodes that hinders such a reaction. The reaction forms Li_3_N with electrodeposited lithium electrodes, thus showing that the presence of a secondary electrolyte interface (SEI) layer does not inhibit the reaction. However, other clean lithium surfaces (e.g., a freshly cut surface) would also be suitable for this treatment. These findings demonstrate that using N_2_ or other Li_3_N-forming additives (e.g., azides^10^) is a highly promising avenue of lithium protection since their capacity to form Li_3_N in situ remains as the battery cycles (which unavoidably produces new lithium surfaces exposed to the electrolyte, thus requiring such protection).

This work combines synchrotron-based X-ray photoelectron spectroscopy (XPS), scanning transmission X-ray microscopy (STXM), and optical microscopy to provide a thorough understanding of the reaction of (electrodeposited) lithium with N_2_(g), as well as the reactions of the thus-formed Li_3_N-protected lithium anodes with the electrolyte. We also investigate the reaction of Li_3_N-coated lithium electrodes with polysulfide-containing solutions, and we show that the Li_3_N coating suppresses the parasitic reaction of polysulfides and other electrolyte species with the lithium electrode. STXM has been used to monitor the morphological and chemical changes of lithium and sulfur electrodes during cycling,^13–16^ but this is the first time in which this technique is employed to directly study the reaction of lithium with polysulfides or the role of Li_3_N protection. In addition, by means of unidirectional (i.e., plating) and bidirectional (i.e., plating and stripping) electrochemical experiments, we show that the Li_3_N coating suppresses lithium dendrite formation and leads to stable cycling due to minimal electrolyte degradation. Overall, the combination of techniques shown here enables a direct probe of the electrolyte degradation reactions of lithium electrodes, with and without Li_3_N coating, thus providing the fundamental understanding required to support the design of suitable coating approaches for the development of lithium metal batteries, including lithium–sulfur.

## 2 Experimental Section

### 2.1 Preparation of Lithium Electrodes and Nitridation

Two sources of lithium were employed in this work. Battery grade lithium foil (Rockwood lithium) was used as received with 11 mm diameter electrodes punched from the foil. Additionally, thin lithium deposits were electrodeposited on nickel discs or nickel TEM grids, as described below.

The electrolyte solution used for the electrodeposition of lithium onto nickel was 4 M LiTFSI (99.95%, Aldrich) in DOL (99.8%, anhydrous, ∼75 ppm butylated hydroxytoluene inhibitor, Aldrich). LiTFSI was dried under vacuum (<0.2 mbar) at 140 °C for 48 h before use. After drying, the LiTFSI salt was stored in an argon-filled glovebox (Unilab, MBraun, H_2_O and O_2_ content <0.1 ppm). DOL was opened in the glovebox and dried over molecular sieves (4 Å, 8–12 mesh bead size, Aldrich, dried under vacuum at 180 °C for 48 h) for 2 days prior to use. The electrolyte solution was prepared in an argon-filled glovebox by dissolving the appropriate amount of LiTFSI into the DOL solvent. Electrolyte solutions were used within 4 weeks, and the water content was <10 ppm, as determined by Karl Fisher titration.

Electrodeposited lithium electrodes were prepared in two-electrode cells [Stainless-Steel Swagelok cell lined with fluorinated ethylene propylene (FEP) film and with two polished Cu current collectors].^17^ A Ni disc (Advent Research Materials 99.8%, 11 mm diameter) was used as the working electrode against a lithium foil counter electrode (Rockwood Lithium, 12 mm diameter). For the characterization by STXM, a Ni transmission electron microscopy (TEM) grid (Agar Scientific, 100 mesh, 3.05 mm diameter) was employed instead of the Ni discs, and for the XPS measurements, a Ni disc of 4 mm diameter was employed. Two Celgard 2400 discs (12 mm diameter, dried under vacuum at 60 °C for 28 h) were used as separators, wetted with 80 μL of the electrolyte. Electrodeposition of lithium onto Ni was performed on a BioLogic MPG multichannel potentiostat. An initial rest period of 30 min was used to record the open-circuit potential. Three plate/strip cycles followed, at a current density of 0.25 mA cm^–2^ with a 5 min rest between each change in the current direction. A final 16 h plate was carried out at 0.5 mA cm^–2^. The cells were then disassembled inside an argon-filled glovebox, washed with DOL to remove any salt, and stored in the glovebox for nitrogen treatment or electrochemical testing (plated lithium electrodes were typically used within 1 week). Dedicated glassware was designed in-house for nitridation of lithium electrodes (Supporting Information, Figure S1), and full details of the procedure are given in the Supporting Information.

### 2.2 Electrochemical Characterization

The electrolyte solution used for unidirectional galvanostatic polarization and lithium plating/stripping measurements was 1 M lithium hexafluoroarsenate (LiAsF_6_) in DOL with 100 ppm TBA stabilizer, as reported previously.^18,19^ LiAsF_6_ (98%, Aldrich) was dried under vacuum (<0.2 mbar) at 140 °C for at least 48 h before use. After drying, the LiAsF_6_ salt was stored in an argon-filled glovebox (Unilab, MBraun, H_2_O and O_2_ content <0.1 ppm) prior to use. TBA (99.5%, Aldrich) was opened in the glovebox and dried over molecular sieves (4 Å, 8–12 mesh bead size, Aldrich, dried at 200 °C for 2 days under vacuum) for 48 h prior to use.

Unidirectional galvanostatic polarization measurements were recorded by using a multichannel potentiostat (VMP2 or MPG, Bio-Logic). Two-electrode Swagelok cells (stainless-steel cell housing lined with FEP copolymer with polished copper current collectors) were constructed with either unmodified (electrodeposited lithium without N_2_ exposure) or nitrided (electrodeposited lithium, nitrogen-treated for 2 h at 90 °C) electrodes against a battery-grade lithium foil (Rockwood Lithium) counter electrode. A gasket (1 mm diameter hole, Viton, fluoropolymer elastomer) was used as a spacer and filled with 15 μL of the electrolyte. A constant negative current was applied to the working electrode (constant plating of Li onto the working electrode), and the potential response vs time was recorded. Great care was taken when disassembling the Swagelok cells as dendritic lithium is extremely reactive to moisture in the air and can ignite. The risk was minimized as the area of dendritic lithium on the electrode was limited to a circle with a 1 mm diameter by the gasket.

Lithium plating/stripping measurements were recorded using galvanostatic cycling with potential limitation (GCPL) settings on a multichannel potentiostat (VMP2 or MPG, Bio-Logic). Symmetric cells (unmodified or nitrogen-treated electrode, 11 mm diameter) were constructed with two Celgard 2400 separators wetted with 80 μL of the electrolyte. Galvanostatic cycling was performed at a fixed current density of 2 mA cm^–2^ over 1.5 h charge (plate) and discharge (strip) intervals for 25 cycles. Electrochemical impedance spectroscopy (EIS) measurements were recorded before and after the plate/strip measurements on a multichannel potentiostat (VMP3, Bio-Logic). A sinusoidal potential with a 10 mV amplitude was applied to the open-circuit voltage (OCV) at frequencies from 1 MHz to 10 mHz, with three points averaged at each frequency.

Galvanostatic cycling of electrodeposited lithium electrodes, both unmodified and nitrided, was carried out using Swagelok 316 stainless-steel union cells of 0.5 in. diameter, lined with FEP film. All cell components were cleaned with ethanol prior to drying at 70 °C in a fan-assisted oven. Cells were assembled inside the argon glovebox with 11 mm diameter electrodeposited lithium (unmodified or with 2 h nitridation) on nickel discs, as described in Section 2.1, followed by two 12 mm GF/F glass microfiber filter separators (Whatman, 0.4 mm thick, 0.7 μm pore size) soaked with 120 μL of 1 M LiTFSI + 0.25 M LiNO_3_ in DOL/DME (1:1 by vol). Lithium nitrate (LiNO_3_, Sigma-Aldrich) was dried at 120 °C under vacuum for 3 days, and 1,2-dimethoxyethane (DME, Sigma-Aldrich) was dried with 3 Å molecular sieves for 3 days. An 11 mm diameter sulfur electrode (1.6 mg_(S)_/cm^2^, Oxis) was placed on top. Cells were allowed to equilibrate at the OCV for 2 h prior to cycling. GCPL measurements employed lower and upper voltage limits of 1.6 and 2.6 V, respectively, at C/10.

### 2.3 Other Characterization Methods

Ex situ STXM measurements of nitrided/unmodified electrodeposited lithium deposits (∼10–50 μm) on nickel TEM grids exposed to polysulfide electrolyte solutions (see Section 2.3) were collected on the I08 beamline at Diamond Light Source (Oxfordshire, UK). 2D optical density images were produced by raster scanning the sample across a focused beam of ∼90 nm spot size and recording the transmitted X-ray intensity. Image stacks were acquired with 0.2 eV energy steps over the main features of the sulfur K edge (2470–2476 eV) and 0.5 eV steps in the energy regions below (2456–2470 eV) and above (2476–2504 eV) the sulfur K edge region. The as-received signals were converted to optical density using incident signal (*I*_0_) measurements from an adjacent empty region of the image above the sulfur K edge. Postexperiment data processing was performed using MANTiS spectromicroscopy and aXis 2000 software.^20^ MANTiS was used to normalize the sulfur K edge spectra and subtract the dark signal. Corrections for spatial drifts in the image stack were performed with an aXis 2000. MANTiS software was further used to perform principal component and cluster analysis (CA), whereby a set of eigenimages and eigenspectra from the data covariance matrix are grouped into clusters of pixels with a similar spectral response. Sulfur standards of LiTFSI and Li_2_S_*n*_ were prepared on carbon film TEM grids.

Ex situ XPS measurements of nitrided/unmodified electrodeposited lithium deposits on 4 mm diameter Ni discs, exposed to the same polysulfide solutions as the STXM samples (see Section 2.3), were collected on the I09 beamline at the Diamond Light Source. One soft incident photon energy (1 keV), selected by *a* plane grating monochromator, was chosen for greater surface sensitivity.^21^ Two further hard excitation energies (2.15 and 6.45 keV), delivered by a Si(111) double-crystal monochromator, were chosen to probe the sample at greater depths. High-resolution spectra were recorded using a hemispherical VG Scienta EW4000 analyzer set to a step size of 0.05 eV. The pass energy was 50 eV for the soft incident photon energy and 200 eV for the two hard incident photon energies.

Additional ex situ XPS measurements of an electrodeposited (unmodified and nitrided) lithium electrode were acquired with a conventional X-ray source using a Kratos Axis Supra. Samples were transported from the glovebox for XPS measurement using the Kratos air-sensitive transfer arm. XPS data were acquired using monochromated Al K_α_ (1486.69 eV) X-rays at 15 mA emission and 15 kV HT. High-resolution spectra were obtained using a pass energy of 20 eV and step size of 0.1 eV. Ar^+^ sputtering was carried out for a total etching time of 30 s.

The software package CasaXPS was used for analysis of photoelectron spectroscopy data, and the Gaussian/Lorentzian peak shape GL(30) was used throughout the fitting procedures. Energy calibration was performed by setting the binding energy for the adventitious carbon (C–C) in C 1s spectra to 285.0 eV, and a Shirley-type function was used for background subtraction. For fitting the S 2p spectra, the ratio of areas of the 2p_1/2_ and 2p_3/2_ peaks was fixed to 0.5. The probing depth of XPS data collected using a synchrotron source was approximated as three times the inelastic mean free path (IMFP), and details for the IMFP calculations are provided in the Supporting Information.

The nitrided/unmodified electrodeposited lithium deposits on nickel TEM grids were also studied with optical images measured with an Olympus BH2 microscope with both light field and dark field imaging. Due to the air-sensitive nature of the samples, they were first sealed inside an argon-filled glovebox into a chamber with glass slide windows before being transferred to the optical microscope for imaging.

### 2.4 Preparation of Polysulfide Solutions

The preparation of lithium polysulfide electrolyte blends followed a procedure previously reported within the group.^22^ In an argon-filled glovebox, lithium sulfide (99.98%, Sigma-Aldrich) and sulfur (S_8_, 100 mesh, sublimed, Sigma-Aldrich, dried at room temperature under vacuum over 72 h) were added into a vial in ratios to give the desired Li_2_S_6_ composition, followed by adding the electrolyte solution 1 M LiTFSI in DOL/DME (1:1 by vol). The solution was stirred at 60 °C for 10 days to ensure full dissolution. After 10 days of stirring, a clear ruby-red polysulfide solution is obtained with an average chain length of Li_2_S_6_.

## 3 Results and Discussion

### 3.1 Synthesis and Characterization of Lithium Nitride Protective Layers

**Figure 1:**
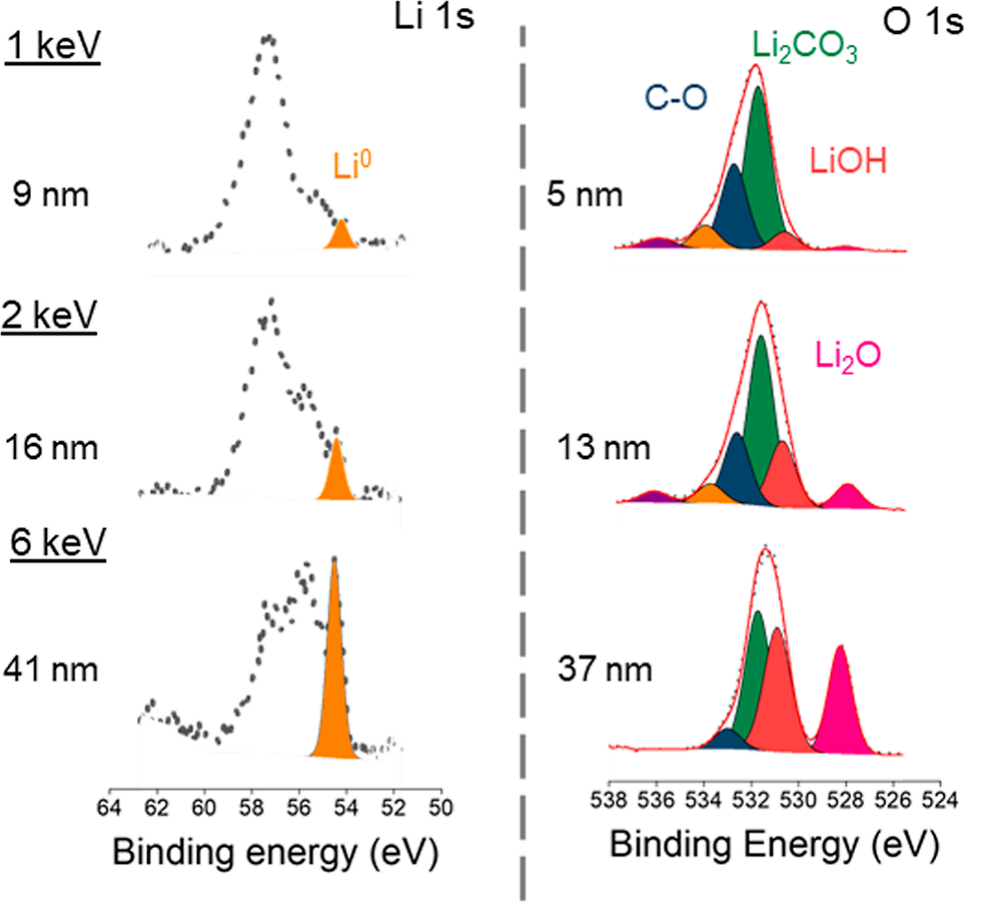
Synchrotron XPS spectra for the Li 1s and O 1s regions of the pristine battery-grade lithium foil as a function of increasing excitation energies, with the calculated average probing depth provided for each spectrum.

The formation of a Li_3_N coating on lithium from reaction with N_2_ gas was first attempted using battery-grade lithium foil (Rockwood Lithium, >99.99% purity). The direct reaction of lithium with N_2_ gas should induce a color change of the metallic lithium surface to a dark red/black of Li_3_N,^10,23,24^ but no obvious color change was observed after 2 h of reaction at 90 °C. XPS analysis of the pristine lithium foil (Figure 1) reveals that it is covered by a mixture of inorganic compounds (Li_2_CO_3_, LiOH, Li_2_O, etc.), forming what has previously been called a native passivation layer.^25–29^ Unfortunately, the properties of the lithium native passivation layer have been shown to be irreproducible due to its high sensitivity toward the storage conditions.^30,31^ Therefore, the present findings show that the direct reaction of N_2_ with lithium metal foil cannot be used reproducibly to form a protective Li_3_N coating, and indeed, under our experimental conditions, such a reaction is suppressed even after 2 h of heating to 90 °C. Below we will show that, fortunately, the reaction proceeds without issues when the lithium foil is replaced by electrodeposited lithium.

The Li 1s core-level spectra of the pristine battery-grade Li foil (Figure 1) contain contributions from multiple lithium-ion compounds that lead to a broad, convoluted peak. However, the metallic Li^0^ signal appears as a separated, sharp peak at 54.5 eV, and it can be used as a reference for estimating when the incident photons are probing the native layer and/or the lithium metal surface underneath.^25,32^ The results in Figure 1 suggest that the native passivation layer is around 10 nm thick since the signal due to metallic lithium is discernible for the lowest excitation energy that gives a probing depth of ∼9 nm in the Li 1s spectrum. In addition, very similar O 1s spectra are observed for the two lowest excitation energies, with probing depths of ∼5 and ∼13 nm, respectively, which again suggests that the passivation layer is around 10 nm thick. Nevertheless, it appears that the passivation layer contains a Li_2_O component that is present deeper within the surface since the intensity of the Li_2_O peak grows when the excitation energy is increased further with a probing depth of ∼37 nm. The details of the assignment of the different peaks are shown in the Supporting Information, Table S3, and Figure S2 shows the C 1s and N 1s spectra.

To improve the reactivity of N_2_ with the lithium metal surface, the lithium foil was replaced by electrodeposited lithium as the target for Li_3_N formation. The electrodeposition protocol involved a preconditioning treatment with three cycles of lithium plating and stripping, followed by a longer lithium plating step of 16 h at a current of 0.5 mA cm^–2^. Repeats of nominally identical cells demonstrate very good reproducibility (Supporting Information, Figure S3). After rinsing the electrode with dry dioxolane, the electrodeposited lithium electrode was exposed to N_2_ for 2 h at 90 °C, and a visible color change was observed during nitridation from a metallic gray of the electrodeposited lithium to a brown-red color indicative of Li_3_N formation on the surface of the lithium electrode (Supporting Information, Figure S4).

**Figure 2:**
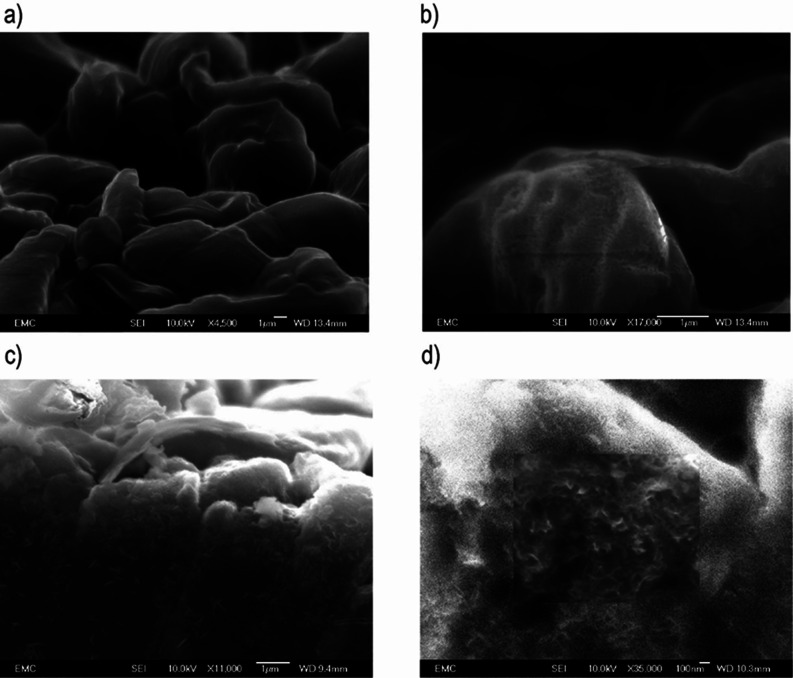
SEM top surface images of unmodified electrodeposited lithium at (a) 4500×, and (b) 17,000× magnification and nitrided electrodeposited lithium at (c) 11,000×, and (d) 35,000× magnification.

Figure 2 presents SEM images of the electrodeposited lithium, as obtained (that is, “unmodified”) and after nitridation with N_2_ (that is “nitrided”). Higher magnification of the nitrided lithium (Figure 2d) reveals sharp edges of material growing at the lithium surface, which could be the formation of Li_3_N crystallites over the metal electrode. The images were also considerably brighter than the SEM images of the unmodified electrodeposited lithium electrode, which could be due to an accumulation of charges on the electronically insulating Li_3_N layer under the electron beam.^23^

**Figure 3:**
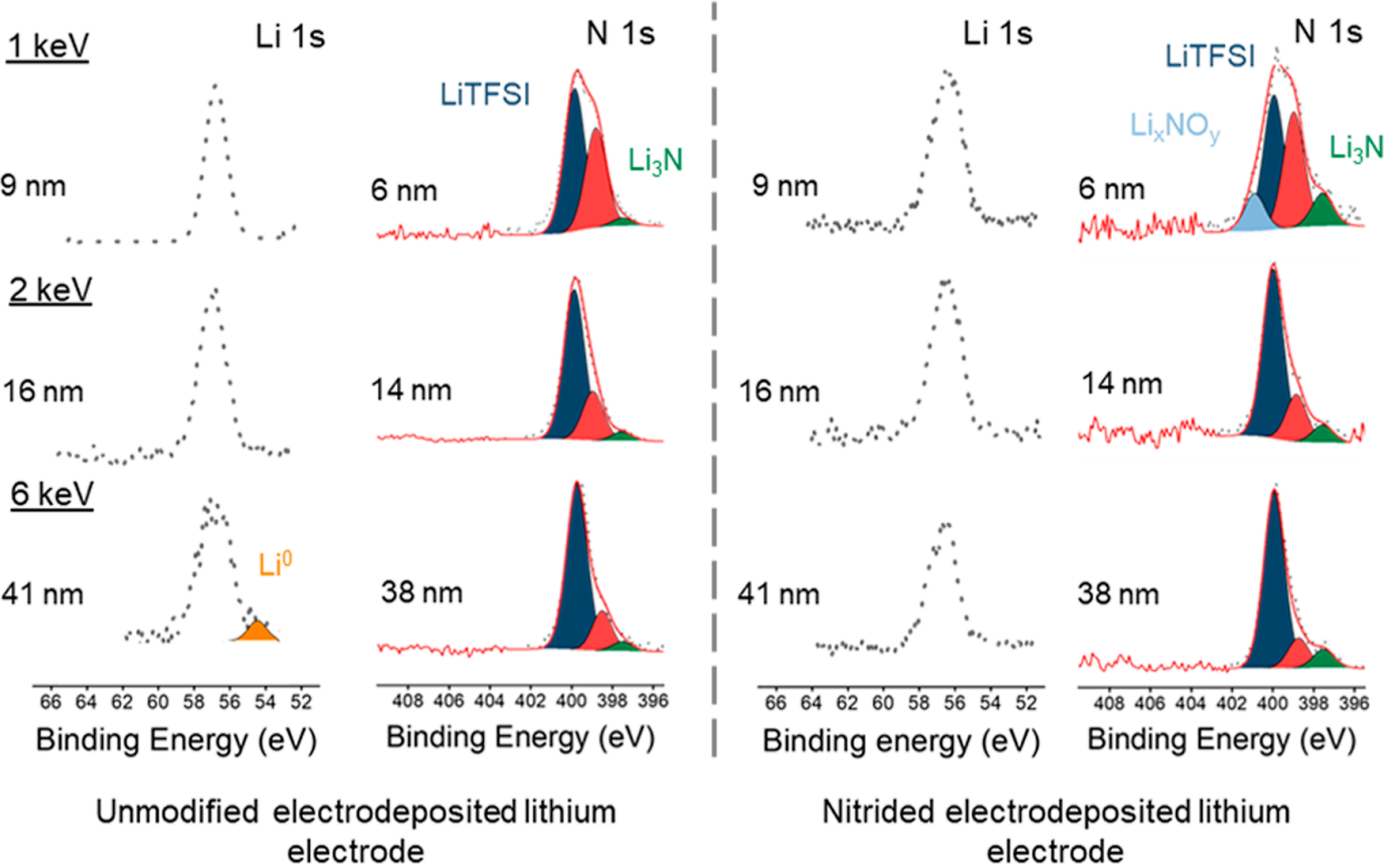
Synchrotron XPS spectra for the Li 1s and N 1s regions of the unmodified (left) and nitrided (right) electrodeposited lithium electrodes as a function of increasing excitation energies, with the calculated average probing depth provided for each spectrum.

Figure 3 presents the Li 1s and N 1s XPS spectra for unmodified and nitrided lithium. Figures S5 and S6 in the Supporting Information show the C 1s and O 1s spectra, and Table S4 lists all the assignments. As the electrodeposited lithium is produced in an electrochemical environment, the surface is covered by an SEI formed by the products of electrolyte decomposition (in this case, 4 M LiTFSI in DOL). Indeed, Figure 3 shows that the Li^0^ signal present at 6 keV (∼41 nm probing depth) is weaker than that for the pristine battery-grade lithium foil, as shown in Figure 1, which suggests that the thickness of the SEI of the electrodeposited lithium is greater than that of the native layer on the battery-grade lithium foil. For the nitrided electrodeposited lithium electrode, the Li^0^ signal is not observable, which suggests that a thicker coating is formed by the nitridation reaction. The fact that the reaction of metallic lithium with N_2_ is hampered by the native passivation layer in the pristine lithium foil, but not by the SEI present in the electrodeposited lithium, can be tentatively ascribed to the more porous structure of the latter,^33–37^ which thus enables N_2_ molecules to reach the underlying metallic lithium.

The N 1s spectra of the unmodified electrodeposited lithium (Figure 3) show a nitrogen environment for the LiTFSI salt (blue) and a LiTFSI decomposition product (red), as well as a weak signal of Li_3_N (green), which is an additional decomposition product of LiTFSI.^38^ Importantly, after nitridation, there is an increase in the relative intensity of the Li_3_N signal in the N 1s spectra at all three incident photon energies (Figure 3). The Li_3_N signal is strongest at the lowest excitation energy (1 keV), as expected for the formation of the Li_3_N coating on the surface of the lithium electrode. The O 1s spectra show a small increase in the contribution of the LiOH/ROLi peak in the nitrided sample (Supporting Information, Figure S6), compared with the unmodified one (Supporting Information, Figure S5). This could be explained by trace water contamination. All other signals are similar for the unmodified and nitrided samples, suggesting that the surface compositions are very similar, apart from the presence of Li_3_N in the latter.

### 3.2 Electrochemistry of Lithium Nitride as a Protective Layer for Lithium Metal Batteries

The previous section demonstrated the successful formation of a Li_3_N coating on electrodeposited lithium metal electrodes upon reaction with N_2_ gas. To determine whether the formed Li_3_N layer improves the electrochemical performance of the lithium metal electrodes, unidirectional galvanostatic polarization and plate/strip measurements were carried out on both the unmodified and the nitrided samples, as shown below.

Unidirectional galvanostatic polarization is an aggressive technique for assessing the failure of lithium metal batteries due to lithium dendrite formation.^39–42^ In this technique, lithium is continuously stripped from one lithium metal electrode and plated onto the other at a fixed current density in symmetric cells. A time to short circuit is recorded when the voltage of the cell tends to zero, indicating that plated lithium has bridged the two metal electrodes. The benefit of testing the cells under these conditions is that there is no break in the deposition (contrary to the case of plate/strip measurements during galvanostatic cycling), and thus, redissolution of lithium dendrites (during the stripping step) is prevented. This electrochemical test thus produces a fast, convenient assessment of the tendency of lithium metal electrodes to form dendrites during electrodeposition (plating).

Since dendrite growth is a stochastic process, the time to short circuit needs to be averaged over many experiments. To improve the reproducibility of the experiment, we constructed cells without a separator between the lithium metal electrodes. In the absence of a physical barrier, plated lithium–dendritic lithium growth can proceed between the two electrodes without restriction, which emphasizes the electrode’s tendency for lithium dendrite growth. This was achieved by replacing the separator with a Viton gasket that had a 1 mm hole in the center, filled with the electrolyte. An electrolyte of 1 M LiAsF_6_ in DOL was chosen for these studies since previous work by Aurbach et al. noted that smooth lithium deposits formed in this electrolyte.^18,19^ One drawback of this electrolyte is the unavoidable presence of arsenic pentafluoride (AsF_5_), formed from the decomposition of the LiAsF_6_ salt.^43^ AsF_5_ is a Lewis acid and can readily polymerize cyclic ethers such as DOL. To stabilize these electrolyte solutions, trace amounts of TBA are added to the solutions as a base to neutralize the Lewis acid in solution, following previous studies.^18,19^

**Figure 4:**
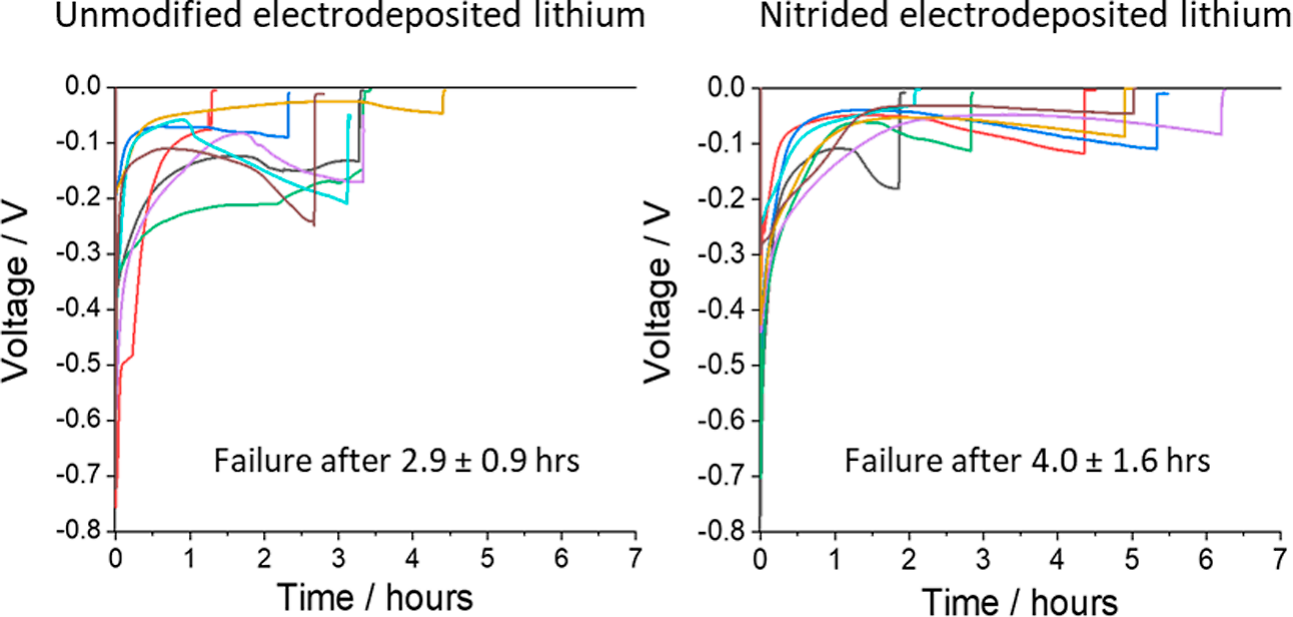
Voltage vs time plots for the unidirectional galvanostatic polarization of the unmodified (left) and nitrided (right) electrodeposited lithium working electrodes in Li–Li cells. Experiments were carried out at a fixed current density of 5 mA cm^–2^, using a pristine battery-grade lithium foil as the counter/reference electrode.

Figure 4 presents the variation of the voltage with time in symmetrical Li–Li cells during unidirectional galvanostatic polarization using either the unmodified or nitrided electrodeposited lithium working electrodes. Eight measurements were recorded for each cell configuration at a fixed current density of 5 mA cm^–2^. It is seen that the average time to short circuit increased from 2.9 ± 0.9 h for unmodified samples to 4.0 ± 1.6 h for the nitrided samples, showing that the nitride layer was effective at preventing lithium dendrite formation. However, significant variations in the cell-to-cell behavior were observed, ascribable to the high deformability of lithium electrodes, whose electrochemical behavior is affected by small indentations or other mechanical defects produced during the manual cell assembly.

**Figure 5:**
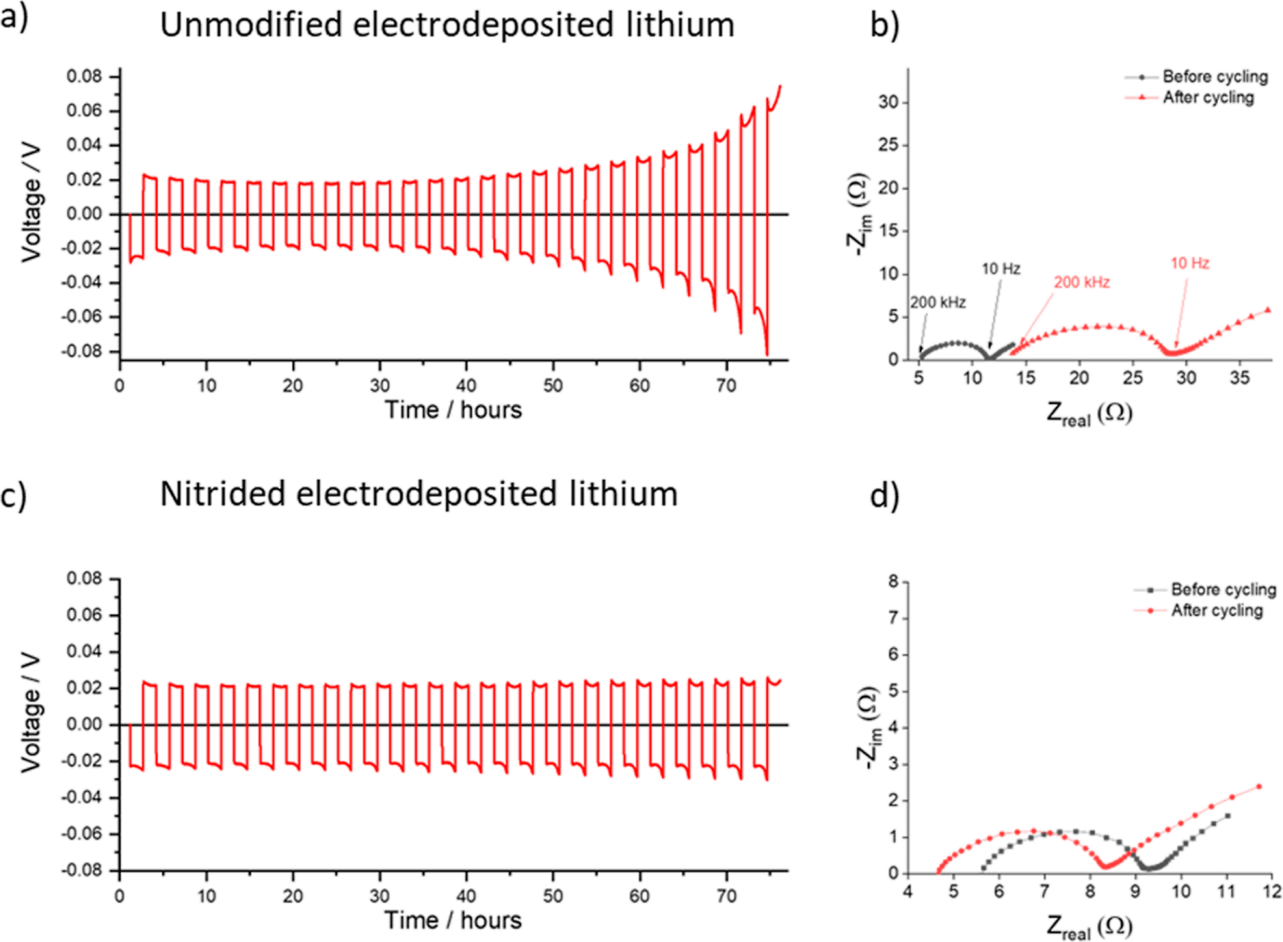
Voltage vs time profiles for the plating/stripping processes of unmodified (a) and nitrided (c) electrodeposited lithium electrodes, using a current density of 2 mA cm^–2^ in Li–Li symmetric cells, and associated Nyquist plots of the impedance spectra for the unmodified (b) and nitrided (d) samples before and after plating/stripping.

The effect of nitridation of lithium electrodes was also investigated by galvanostatic cycling (that is, with a sequence of plating and stripping steps) in Li–Li symmetric cells. The lithium plating and stripping processes were performed at a fixed current density of 2 mA cm^–2^ to a capacity of 3 mA h cm^–2^ for 25 cycles, and EIS was recorded before and after the plate/strip cycles, as shown in Figure 5.

Figure 5 shows that while the unmodified and nitrided lithium electrodes in symmetrical cells show the same polarization at the beginning of galvanostatic cycling, with overpotentials of around 0.02 V. As cycling progresses, the unmodified electrodes develop a higher polarization, reaching overpotentials of around 0.08 V for lithium plating and stripping after 25 cycles, whereas the nitrided lithium electrodes remain stable. Indeed, the impedance measurements show a marked increase in the high-frequency resistance for the cell with unmodified electrodes, whereas the impedance of the cell with the nitrided electrodes shows little change. Previous studies have shown that the increase in the high-frequency resistance is due to surface reactions of lithium electrodes with the electrolyte,^44,45^ and the present results thus show that such reactions are minimized by the presence of a Li_3_N coating in the nitrided lithium sample.

### 3.3 Chemical Stability of Lithium Nitride as a Protective Layer for Lithium–Sulfur Batteries

The previous section demonstrated that Li_3_N acts as an efficient protective coating on lithium metal electrodes that prevents lithium dendrite formation and enables stable lithium plating and stripping by minimizing the electrolyte degradation reactions. In this section, we investigate the suitability of the protection of lithium electrodes with Li_3_N for applications in Li–S batteries. For that purpose, we exposed the lithium electrodes, unmodified and nitrided, to electrolyte solutions containing polysulfides. Specifically, a solution containing 1 M Li_2_S_6_ and 1 M LiTFSI in 1:1 DOL/DME was employed since it represents a common electrolyte used in Li–S batteries, and the polysulfide Li_2_S_6_ was found to be highly soluble in previous studies.^46^

The chemical reaction of lithium electrodes with the polysulfide containing electrolyte was monitored by STXM, optical microscopy, and XPS. For the microscopy characterization, the electrodeposition of lithium was carried out onto nickel TEM grids, using the same experimental conditions as described above, except for the fact that the final plating time was reduced to 15 min, to produce thin lithium deposits of ∼10–50 μm of thickness, suitable for STXM transmission measurements (Supporting Information Figure S7).

**Figure 6:**
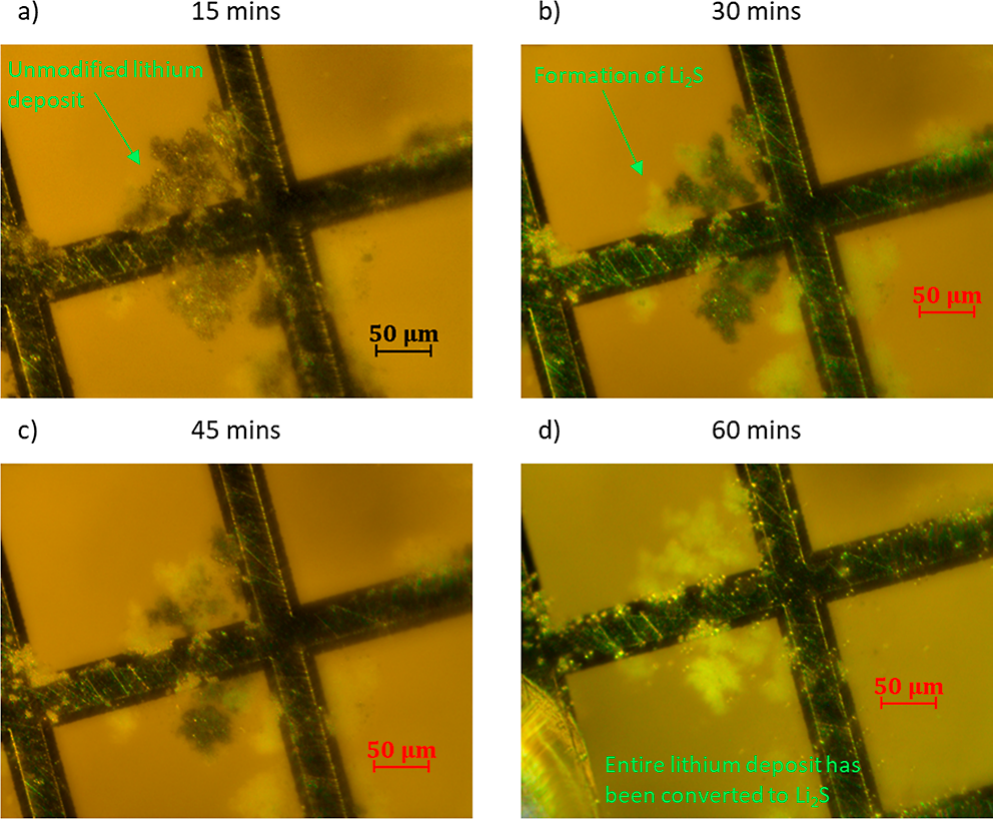
Series of dark-field optical microscopy images of an unmodified electrodeposited lithium electrode exposed to a polysulfide solution after 15 (a), 30 (b), 45 (c), and 60 min (d).

Figure 6 presents optical microscopy images of the unmodified electrodeposited lithium deposits on the nickel TEM grid after different times of exposure to the polysulfide solution. Significant conversion of the metallic lithium deposits into white Li_2_S precipitates is evident after 30 min of polysulfide exposure, and the whole metallic lithium deposit appears to be converted into Li_2_S after 60 min. The formation of solid Li_2_S is confirmed by STXM, see below, and the overall reaction can be summarized as follows
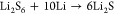


**Figure 7:**
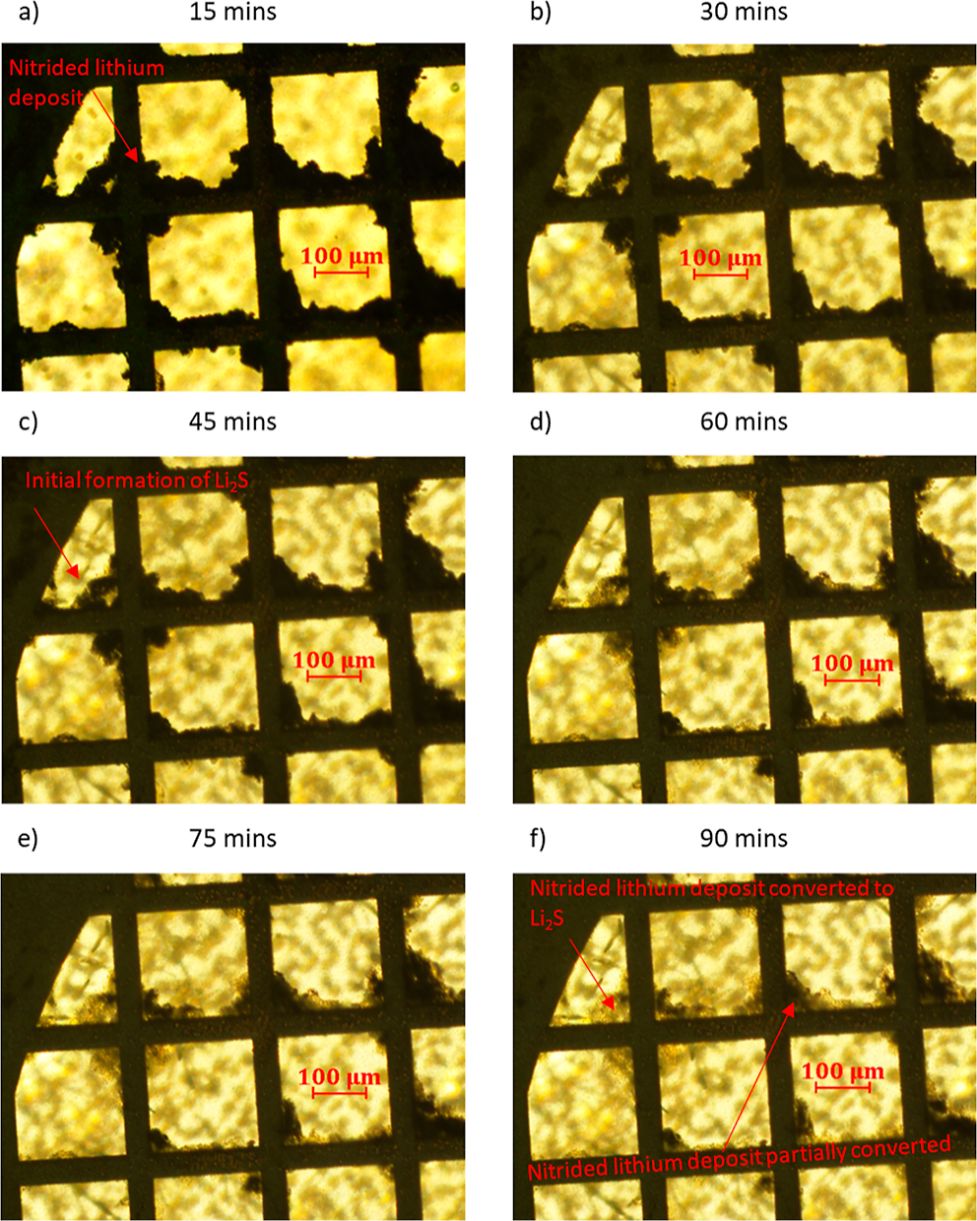
As shown in Figure 6 but with the nitrided electrodeposited lithium electrode exposed to a polysulfide solution after 15 (a), 30 (b), 45 (c), 60 (d), 75 (e) and 90 min (f).

The same experiments were carried out with electrodeposited lithium electrodes that had been nitrided in N_2_ gas, and the corresponding optical images during exposure to the polysulfide solution are shown in Figure 7. In contrast to the results with the unmodified electrodes, in the case of the nitrided sample, the formation of Li_2_S is much slower and partial, and the full conversion of metallic lithium to Li_2_S is still not complete after 90 min of exposure. These results evidence that although the presence of Li_3_N in the nitrided sample does not prevent the formation of Li_2_S from polysulfide reduction, it slows down the reaction rate.

**Figure 8:**
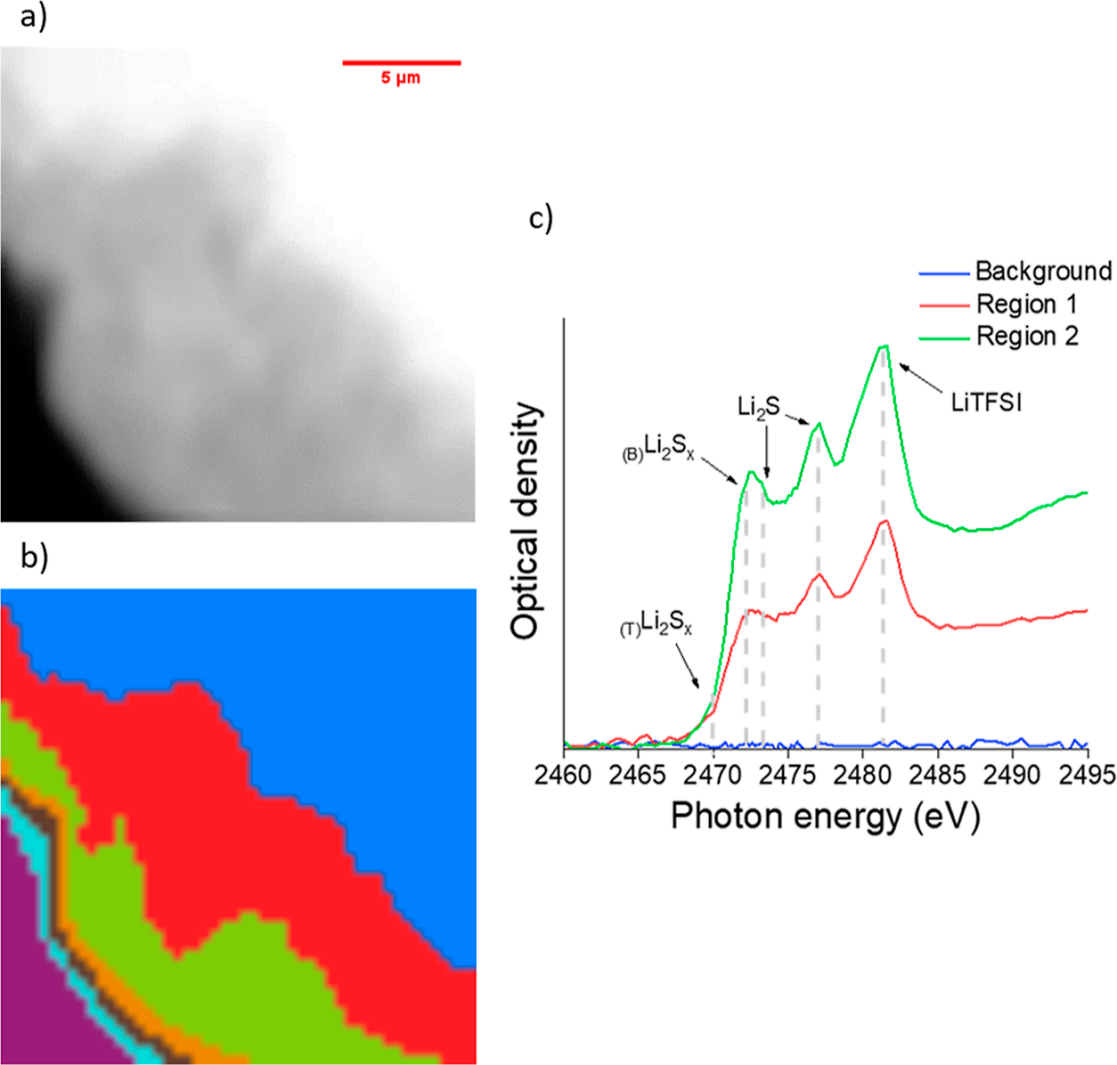
(a) STXM image (15 × 15 μm, 2460 eV, pixel size = 150 nm) of an unmodified electrodeposited lithium electrode exposed to a polysulfide solution for 30 min, (c) near-edge X-ray absorption fine structure (NEXAFS) spectra at the S K-edge energies, and (b) corresponding cluster map.

**Figure 9:**
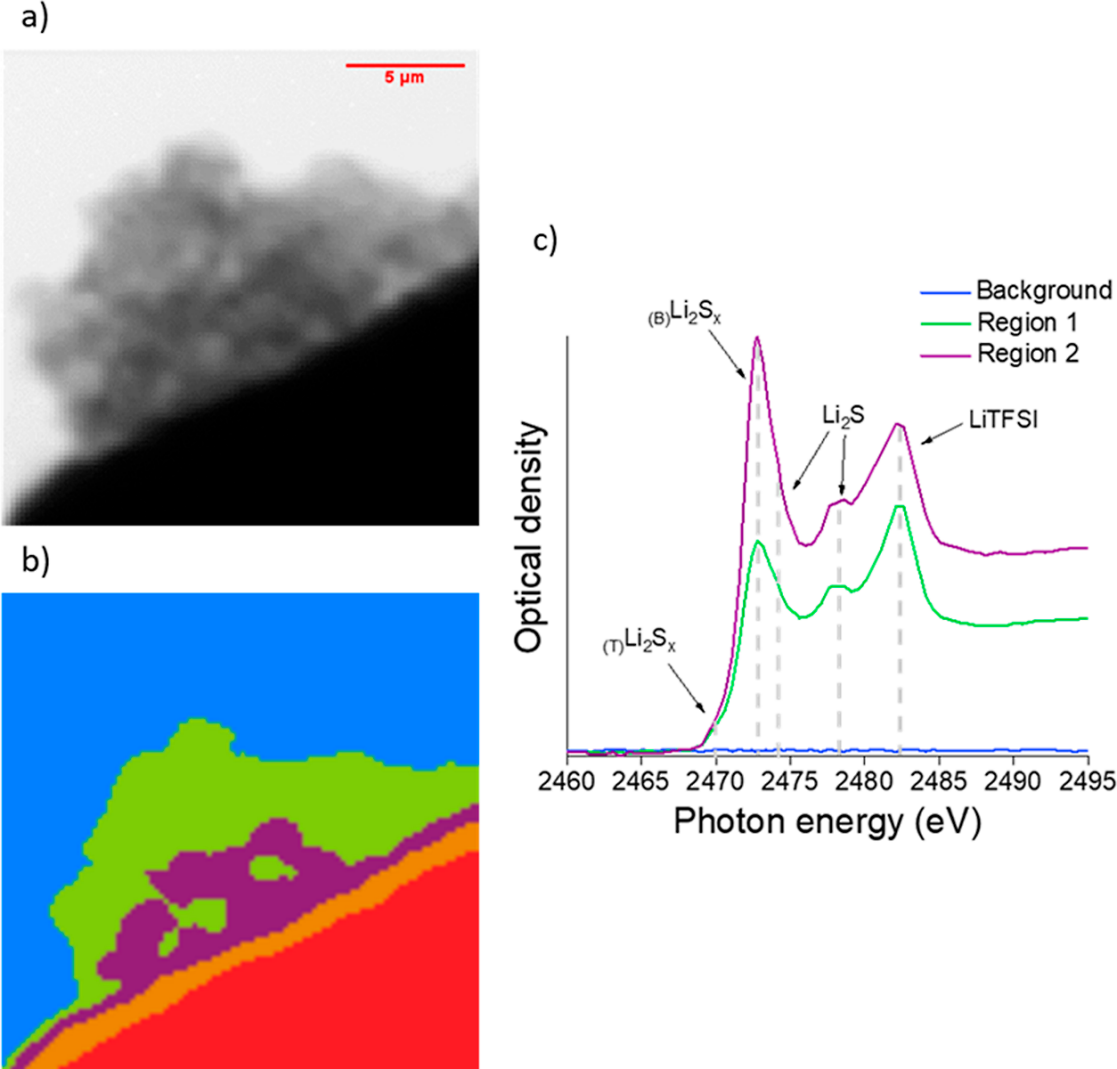
As shown in Figure 8 but for a nitrided lithium electrode.

To directly probe the reaction between lithium and polysulfides, chemical analysis of the unmodified and nitrided electrodeposited lithium was carried out, after exposure to the polysulfide solution, by STXM and XPS. An exposure time to the polysulfide solution of 30 min was selected based on the optical microscopy results (Figures 6 and 7), in which extensive conversion to Li_2_S was observed for the unmodified sample, whereas marginal conversion was observed for the nitrided one. After exposure to the polysulfide solution, the samples were washed with dioxolane to prevent further reactions. The results of the analysis of the STXM results for the unmodified and nitrided samples are shown in Figures 8 and 9, respectively. To aid in the interpretation of the results, standard spectra for LiTFSI and Li_2_S_6_ were collected (Supporting Information, Figure S8).

Figure 8 shows that the unmodified lithium deposit, exposed to the polysulfide solution, exhibits three main regions, as identified by CA of the stacked STXM images. The background (blue) shows no absorption and corresponds to the area that had no lithium deposits. Regions 1 (red) and 2 (green) represent two main regions of the lithium deposit, which show the same absorption features but with different intensities. Absorption by polysulfide species (Li_2_S_*x*_) was observed at 2470 eV (pre-edge) and 2472 eV for the terminal (S 1s to Li–S σ*) and bridging (S 1s to S–S π*) Li_2_S_*x*_ environments, respectively, in agreement with the results from the Li_2_S_6_ standard (Supporting Information, Figure S8). Additionally, a strong absorption at 2476.5 eV is due to the presence of lithium sulfide (Li_2_S) and is assigned to the S 1s to Li_2_S σ* transition.^47–49^ The absorption at 2482.5 eV is attributed to the S 1s to S=O π* transition in the LiTFSI salt, as recorded from the LiTFSI salt standard (Supporting Information, Figure S8). Interestingly, the relative intensities between the absorption features due to polysulfide species and Li_2_S remain constant throughout the lithium deposit, suggesting that the chemical composition is the same and that the only difference is the amount of material present, which is lower in region 1 (red) compared to region 2 (green).

For the nitrided sample (Figure 9), CA also identifies three main regions. Again, the background (blue) shows no absorption, as expected for the empty region. Regions 1 (green) and 2 (purple) represent two main regions within the nitrided lithium deposits. Li_2_S was observed in both regions, but the relative intensity was lower with respect to the polysulfide species (Li_2_S_*x*_), when compared with the unmodified lithium deposit (Figure 8). The innermost region (region 2, purple) contains a strong absorption feature due to Li_2_S_*x*_, suggesting that Li_2_S formation proceeded at a very slow rate. Overall, the presence of Li_2_S in the nitrided lithium sample after polysulfide exposure is only minor, thus evidencing that the Li_3_N coating has suppressed the parasitic reaction of polysulfides that consumes Li metal and forms Li_2_S.

XPS measurements were then carried out to obtain a more detailed chemical analysis of the composition of the unmodified and nitrided lithium electrodes after exposure to the polysulfide solution. As for the STXM, the electrodes were immersed in the polysulfide solution for 30 min, followed by washing in DOL. The XPS measurements were carried out with electrodeposited lithium electrodes deposited on 4 mm diameter nickel discs, using a final plating time of 16 h.

**Figure 10:**
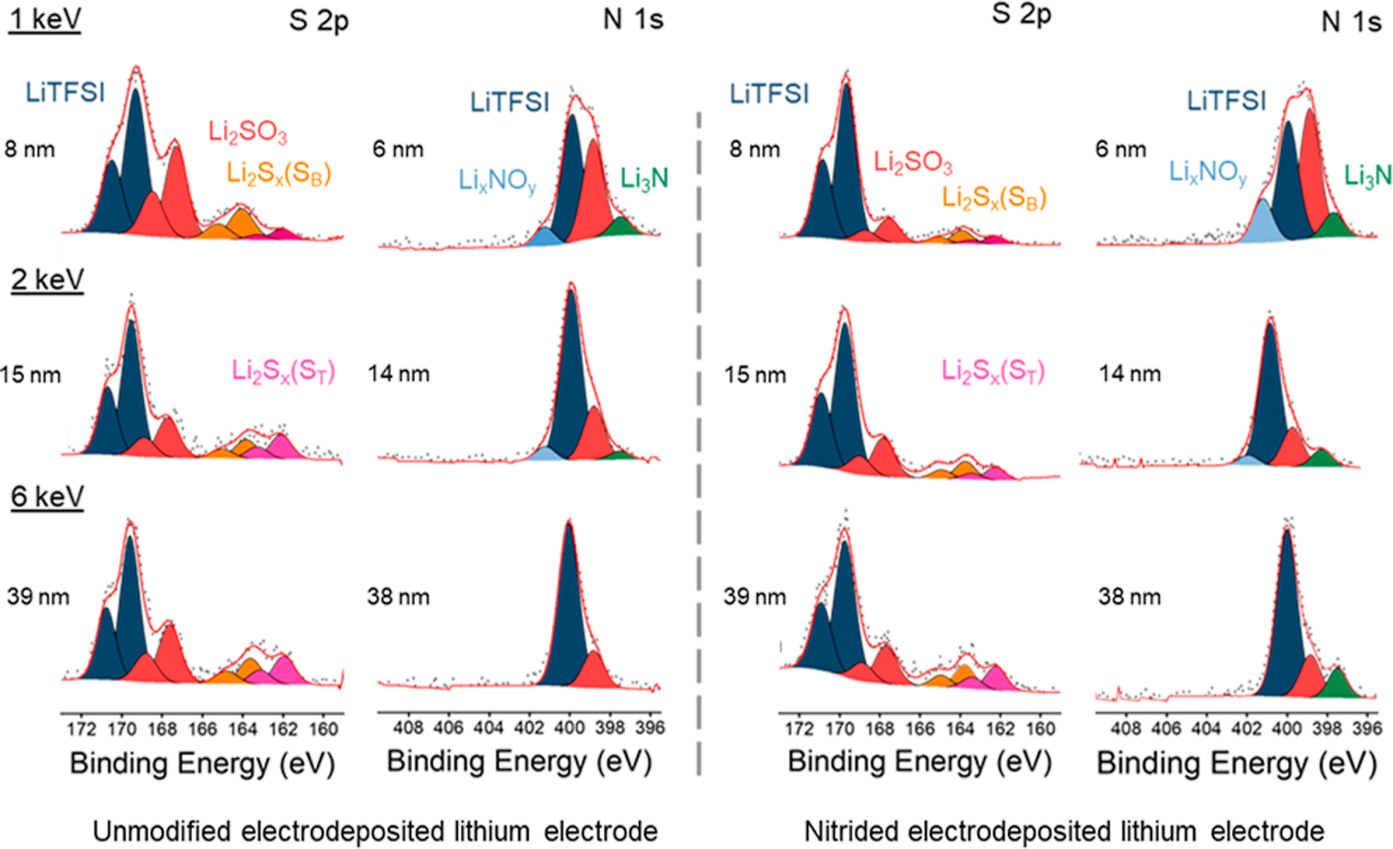
Synchrotron XPS spectra for the S 2p and N 1s regions of the unmodified (left) and nitrided (right) electrodeposited lithium electrodes exposed to a polysulfide solution for 30 min, as a function of increasing excitation energies, with the calculated average probing depth provided for each spectrum.

Figure 10 presents the N 1s and S 2p XPS spectra, and Table S5 in the Supporting Information summarizes the assignment of the core levels. For both the unmodified and nitrided electrodeposited lithium electrodes, the N 1s spectra are very similar to those of the electrodeposited lithium electrodes without exposure to the polysulfide solution (Figure 3). Importantly, the Li_3_N value remains the same, which shows that polysulfides do not consume the Li_3_N coating. The S 2p XPS spectra show that the main doublet is due to the LiTFSI salt, followed by a doublet ascribable to sulfite (Li_2_SO_3_) species formed from decomposition reactions of polysulfides with LiNO_3_ at lithium electrodes.^38,50–52^ Interestingly, the Li_2_SO_3_ peaks are more intense for the unmodified samples, which suggests that the nitrided sample has suppressed polysulfide degradation, due to the Li_3_N coating protection. Finally, two doublets of S 2p peaks are observed at lower binding energies, ascribable to bridging sulfur in S–S covalent bonds (S_B_) and terminal sulfur (S_T_), respectively.^53–55^ For the unmodified sample, the intensity of the terminal sulfur increases at the expense of the intensity of the bridging sulfur of polysulfides as the probe penetration depth increases, which can be ascribed to the conversion of long-chain polysulfides to short-chain polysulfides at depths closer to the reactive lithium surface, in agreement with previous studies.^53,54,56^ On the other hand, for the nitrided sample, the intensity of the bridging and terminal sulfur is very small and only clearly discernible for relatively large probe depths (∼39 nm), which could be due to small amounts of polysulfides trapped inside small pores of the lithium electrode. In addition, for the nitrided sample, the conversion of long-chain polysulfides to short-chain polysulfides is not observed, which suggests that such a reaction, which is undesirable because it corrodes the lithium electrode, is suppressed by the Li_3_N coating.

**Figure 11:**
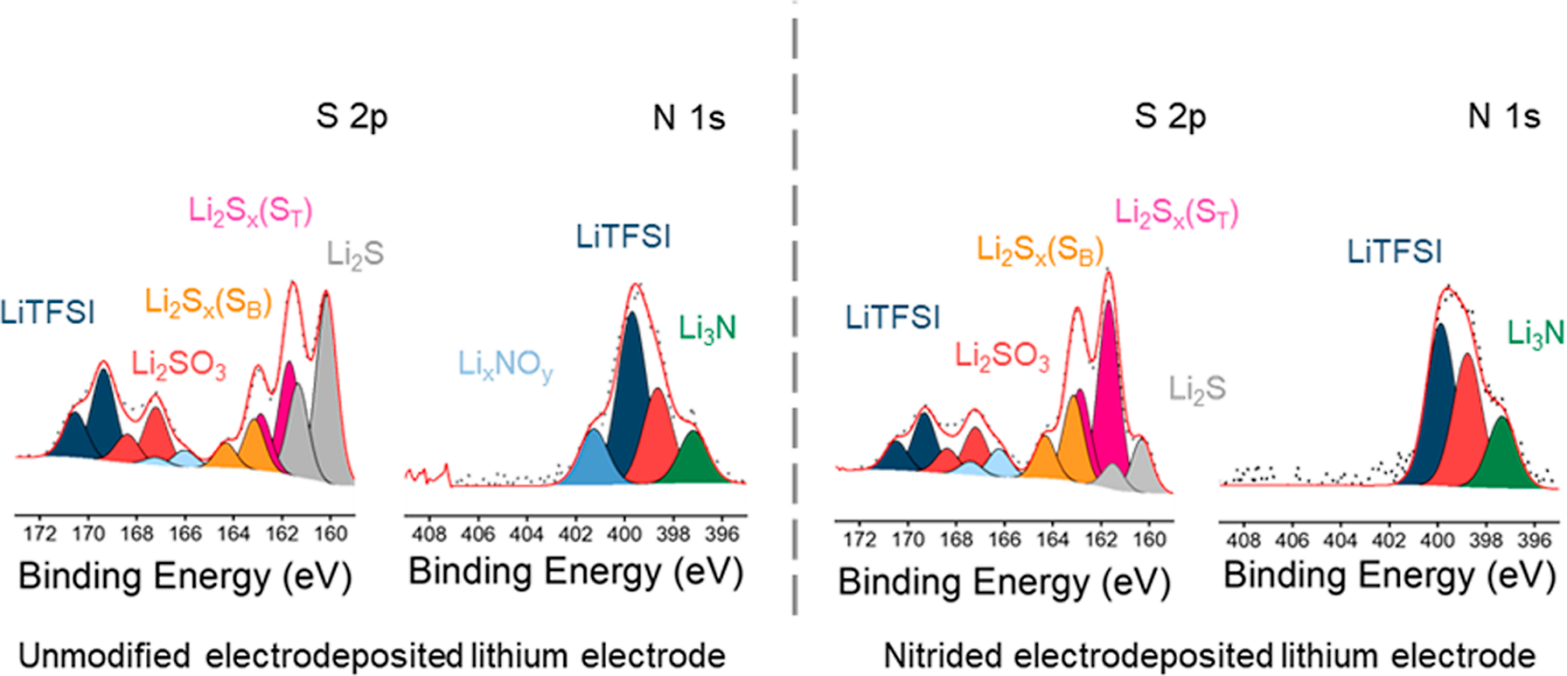
XPS spectra (conventional Al K_α_) for the S 2p and N 1s regions of the unmodified (left) and nitrided (right) electrodeposited lithium electrodes exposed to a polysulfide solution for 30 min, after 30 s of etching.

Additional XPS measurements were carried out using a conventional X-ray source (Al K_α_), after 30 s of Ar^+^ etching, to probe further into the composition of the surface region of the electrodeposited lithium (unmodified and nitrided) electrodes exposed to the polysulfide solution. Figure 11 presents the N 1s and S 2p XPS spectra, and Tables S6 and S7 in the Supporting Information summarize the assignment of the core levels and the results of the quantification analysis, respectively.

The S 2p XPS spectra of the lithium electrodes in Figure 11 reveal the presence of Li_2_S, which is the expected final reaction product of the reduction of polysulfides by the lithium metal. The absence of Li_2_S in the synchrotron XPS spectra in Figure 10 is ascribed to the insufficiently deep probing depth in these experiments since Li_2_S is expected to form at the buried interface between the SEI and the underneath lithium metal surface. Importantly, the results in Figure 11 show that the Li_2_S peaks are greatly reduced in the spectra of the nitrided lithium electrode compared to the unmodified one, thus further corroborating that the nitridation treatment has suppressed/mitigated the parasitic reduction of polysulfide by lithium.

Finally, the effect of nitridation of lithium metal anodes was tested on Li–S batteries, which were assembled with electrodeposited lithium electrodes that were either unmodified or nitrided (Figures S9 and S10, Supporting Information). The results with the nitrided electrodes show better capacity retention and Coulombic efficiency, which again can be ascribed to the suppression of parasitic reactions.

## 4 Conclusions

We have shown that a protective Li_3_N coating on lithium electrodes can be formed from the reaction of lithium metal with N_2_ gas provided that the lithium metal is free from a passivation layer. Unfortunately, battery-grade lithium foil, commonly used in battery research laboratories, unavoidably presents a native passivation layer, which prevents the reaction with N_2_. However, lithium electrodes formed via electrodeposition successfully react with N_2_ to form a protective Li_3_N coating, despite the presence of a SEI formed during the electrodeposition process. The formation of the Li_3_N coating is confirmed by N 1s XPS analysis, and it is further corroborated by the visible change in the color of the lithium electrode from metallic to brown-red.

Interestingly, the thus-formed Li_3_N coating on an electrodeposited lithium electrode produces significant improvements in the electrochemical performance. Through unidirectional constant current measurements, the time to short-circuit due to dendrite formation in Li–Li cells is seen to increase from 2.9 ± 0.9 to 4.0 ± 1.6 h, when comparing repeats of multiple cells with lithium electrodes without and with the Li_3_N coating, respectively. Repeated constant current plating and stripping measurements also show visible improvements with Li_3_N-coated electrodes, which show stable behavior, whereas for the uncoated ones, an increase in polarization with cycling is observed, which is caused by an increase in the high-frequency resistance, as measured by impedance, which in turn is caused by electrolyte degradation reactions on the uncoated lithium electrodes.

The Li_3_N coating also produces significant improvements in the stability of lithium electrodes immersed in polysulfide-containing solutions, which are critical for the performance of Li–S batteries. Optical microscopy images show that for thin (ca. 10–50 μm) lithium deposits, full conversion of lithium metal to Li_2_S, upon reaction with a 1 M Li_2_S_6_ solution, takes place in around 60 min, whereas for the Li_3_N-coated deposits, the formation of Li_2_S is not complete after 90 min. Indeed, STXM measurements show that after 30 min of immersion time in the polysulfide solution, the uncoated lithium electrode contains significant amounts of Li_2_S, whereas the signal due to Li_2_S is barely discernible for the Li_3_N-coated electrode. These conclusions are further corroborated by S 2p XPS measurements, which show that the Li_2_SO_3_ signal (due to polysulfide degradation) is largely suppressed for the Li_3_N-coated lithium electrode, as compared to the uncoated one, and that the signals due to polysulfides/Li_2_S also have much smaller intensity. Interestingly, for the uncoated lithium electrode, a conversion of long-range polysulfides to short-range polysulfides, at the surface layers closer to the metallic lithium (that is, at increasing XPS probe depth), is evident from the change in the relative intensities of the bridging and terminal sulfur signals. Such conversion is not seen for the Li_3_N-coated electrode, which suggests that the latter has suppressed reactivity toward such parasitic polysulfide conversion reactions. XPS measurements recorded after Ar^+^ etching reveal a strong Li_2_S pair of peaks in the S 2p spectra of the unmodified electrodeposited electrode. These are noticeably reduced with the nitride electrode, which further confirms the protective role of the Li_3_N coating. Finally, the capacity fade and Coulombic efficiency of Li–S cells also improve when the electrodeposited lithium metal anode has been nitrided, compared to the untreated one.

In summary, the Li_3_N coating on lithium is found to be advantageous to suppress the undesirable reactions of lithium dendrite formation and polysulfide parasitic reduction, and the combination of STXM, XPS, optical microscopy, and electrochemical characterization is found to be very powerful to directly probe these reactions. The formation of Li_3_N is achieved using, for the first time, the direct reaction of electrodeposited lithium electrodes with N_2_ gas, showing that the presence of an SEI on lithium does not prevent such reaction. Hence, the in situ generation of Li_3_N coatings inside the batteries using SEI-covered lithium is feasible and could be studied with the methodology developed here.

## Supporting Information Available

Experimental procedure of the nitridation reaction, description of the method followed for the estimation of probing depths, additional results of the XPS characterization, electrochemical results of repeat cells of the electrodeposition process, photographs of electrodeposited lithium electrodes before and after nitridation, optical images of the electrodeposited lithium on Ni grid, NEXAFS measurements of reference samples, assignments of XPS peaks, and results of the electrochemical characterization of Li–S batteries (PDF)

## Notes

Raw data used in the preparation of this article are available from the University of Southampton repository at https://doi.org/10.5258/SOTON/D2733.
