# Supplementary material for: Combined Electrochemical, XPS, and STXM Study of Lithium Nitride as a Protective Coating for Lithium Metal and Lithium–Sulfur Batteries
Source: ACS Appl Mater Interfaces. 2023 Aug 8;15(33):39198–210. doi: 10.1021/acsami.3c04897 (PMC10450643; doi:10.1021/acsami.3c04897)
Supplement: Supplementary file 1 — am3c04897_si_001.pdf [file am3c04897_si_001.pdf]

Supporting information

A combined electrochemical, XPS and STXM study of lithium nitride as a protective coating for lithium-metal and lithium-sulfur batteries

Samuel D.S. Fitch[a], Gilles E. Moehl[a], Nina Meddings[a], Sacha Fop[a], Samantha Soulé[a], Tien-Lin Lee[b], Majid Kazemian[b], Nuria Garcia-Araez[a]* and Andrew L. Hector[a]*

a: School of Chemistry, University of Southampton, Southampton SO17 1BJ, UK.

b: Diamond Light Source Ltd, Diamond House, Harwell Science and Innovation Campus, Didcot, Oxfordshire OX11 0DE, UK

* Corresponding author (Nuria Garcia-Araez: N.Garcia-Araez@soton.ac.uk.uk, Andrew L. Hector: A.L.Hector@soton.ac.uk)

Procedure for the nitridation of lithium electrodes

The glassware for the nitridation of lithium electrodes has two dedicated gas lines for N_2 or Ar which are controlled by Young's taps that join a single inlet alongside a vacuum line, see Figure S1. An introductory column filled with regenerated molecular sieves is attached to the inlet and can also be individually isolated. The portable reaction chamber houses a copper stage to improve heat transfer to the samples. The chamber was designed to be able to enter a glovebox under vacuum and exit the glovebox filled with argon. The reaction chamber is attached to the introductory column for evacuation and refilling of gases. The dedicated glassware was designed to prevent the exposure of lithium electrodes to air/water.

For the nitridation of lithium electrodes, the electrodes were loaded onto a copper stage inside an argon-filled glovebox (MBraun, oxygen and water content <0.1 ppm) and were transported under argon in the sealed portable reaction chamber. The reaction chamber was attached to the introductory column filled with molecular sieves (dried at 200 °C, 24 h) under a positive flow of argon (BOC Pureshield, 99.998%), the reaction chamber was then immersed in an oil bath at 90 °C for the duration of the nitridation reaction. The entire system was then evacuated for 20 minutes to remove the argon gas. Nitrogen (BOC, 99.998% minimum) gas was carefully backfilled to first fill the introductory column where trace water was removed by molecular sieves. The flow of N_2 was then set to 50 cm^3 min^{-1} to keep a small positive overpressure inside the glassware to prevent contamination from the laboratory atmosphere. N_2 was then backfilled into the heated reaction chamber for a reaction time of 2 hours, afterwards the entire system was again evacuated for 20 minutes to stop the nitridation reaction. The evacuated reaction chamber was reopened inside an argon-filled glovebox, where the nitrogen-treated lithium electrodes were stored for electrochemical testing or characterization (electrodes were typically used within 1 week).

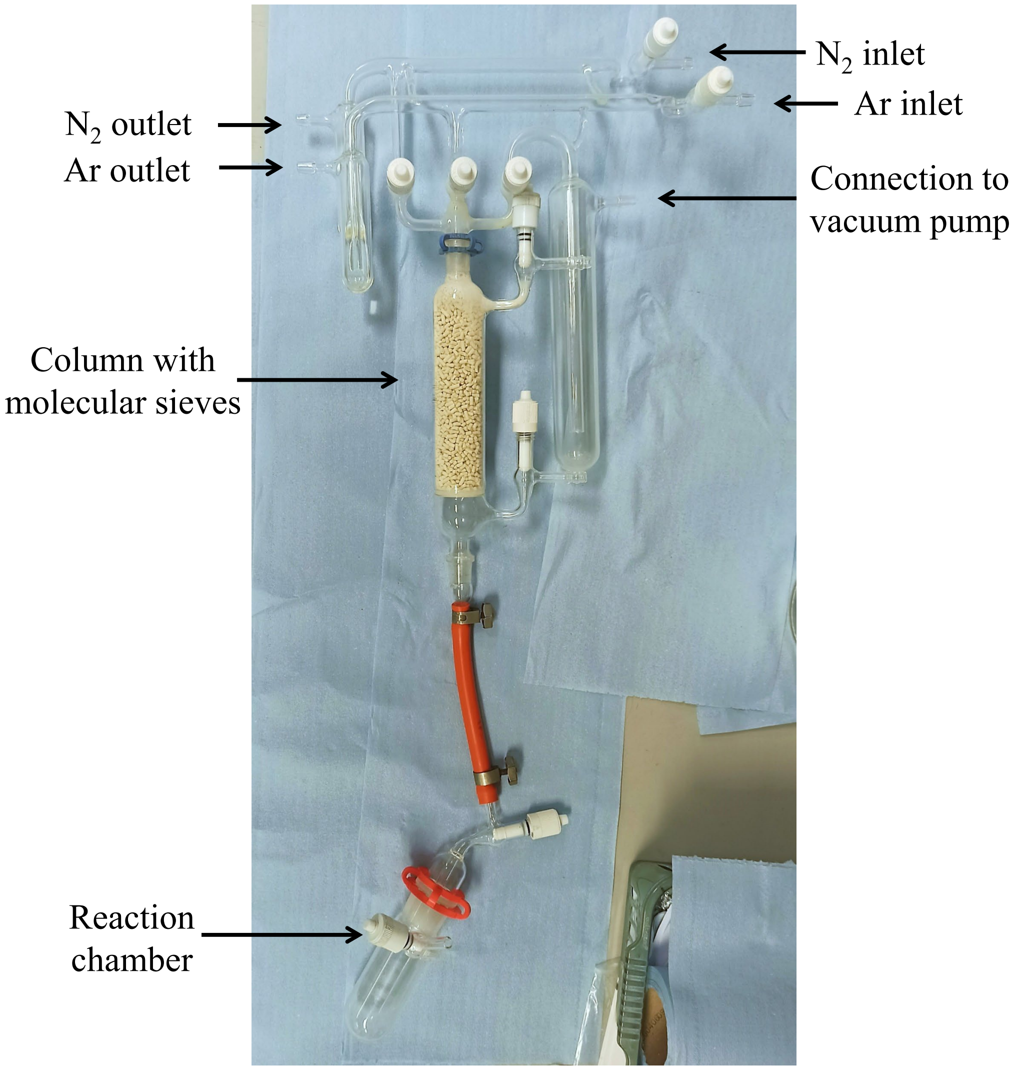

Figure S1 - Dedicated glassware for the nitridation of lithium electrodes.

Calculation of XPS probing depth values

Probing depths were approximated as three times the photoelectron inelastic mean free paths (IMFP), where the latter were estimated from the TPP-2M model developed by Tanuma et al.[1] This model uses four parameters: the molecular weight, the density, the number of valence electrons per formula unit and the bandgap energy. The bandgap is unknown for some compounds, but this parameter has a small influence on the final result, as shown previously.[2]

This definition of the probing depth thus equals the depth from which 95% of the photoelectrons are emitted from a homogeneous material (since the electron take off angle was 90°). The IMFP values are calculated for the main compounds that are detected on the surface, as listed in Table S1. The average probing depths are then calculated using the average IMFP values, and the results are shown in Table S2.

Table S1. Summary of photoelectron inelastic mean free paths (IMFP) values, in angstrom, for different compounds detected on the lithium surfaces, as a function of the incident photon excitation energy.

	**IMFP values, in angstrom, as a function of the excitation energy**		
	**1 keV**	**2.15eV**	**6.45eV**
**Li_2SO_3 (Li 1s)**	26	50	124
**Li_2CO_3 (Li 1s)**	29	53	134
**Li_2O (Li 1s)**	26	49	123
**Li metal (Li 1s)**	33	63	161
**LiOH (Li 1s)**	29	54	135
**LiTFSI (Li 1s)**	31	59	149
**Li_2CO_3 (O 1s)**	17	43	125
**Li_2O (O 1s)**	16	40	115
**LiOH (O 1s)**	17	44	127
**Li_2SO_3 (S 2p)**	24	48	122
**LiTFSI (S 2p)**	29	57	147
**Li_2S (S 2p)**	23	45	117
**Li_3N (N 1s)**	18	41	114
**LiTFSI (N 1s)**	22	51	142

Table S2. Summary of average probing depths, in nm, as obtained from the average of the photoelectron inelastic mean free paths (IMFP) values in table S1.

	**Probing depth values, in nm, as a function of the excitation energy**		
	**1 keV**	**2.15eV**	**6.45eV**
**Li 1s**	9	16	41
**S 2p**	8	15	39
**N 1s**	6	14	38
**O 1s**	5	13	37

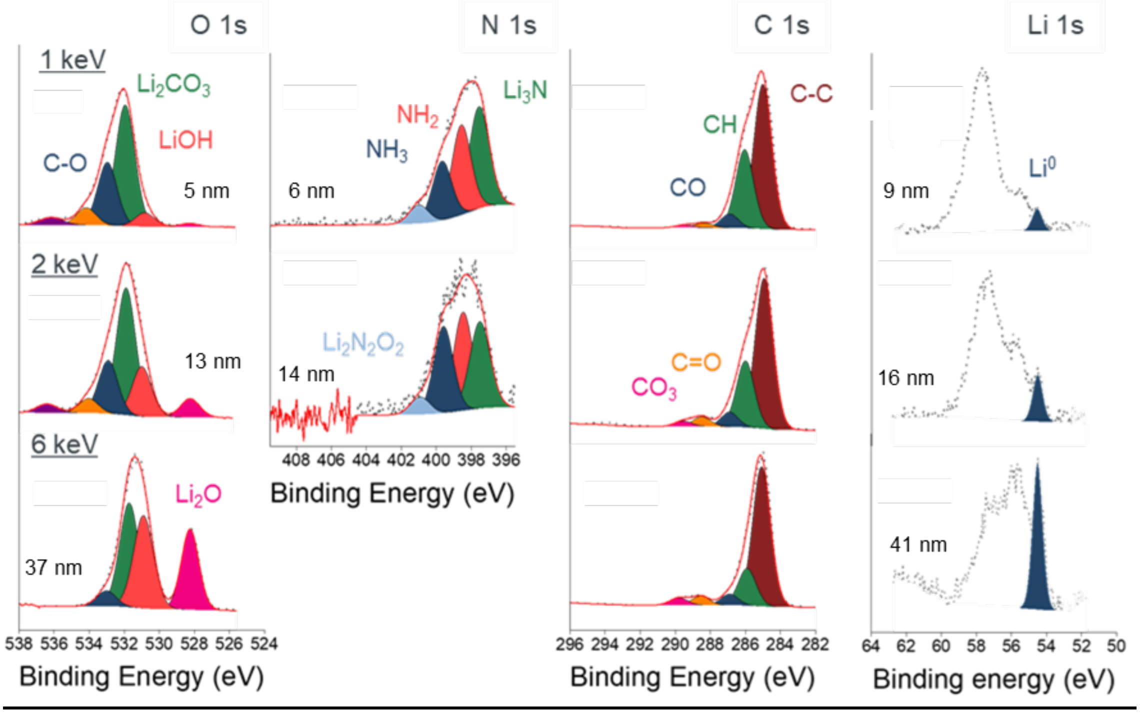

Figure S2. Synchrotron XPS spectra for the O 1*s*, N 1*s*, C 1*s* and Li 1*s* regions of the surface of the pristine battery-grade lithium foil with native passivation layer as a function of increasing excitation energies, with the calculated average probing depth provided for each spectrum.

Table S3. Summary of XPS analysis and assignments for the surface characterisation of the battery-grade lithium foil with native passivation layer shown in Figure S2.

Orbital	Binding energy (eV)	FWHM	Peak assignments
**O 1*s***	528.3	1.2	Li_2O[3,4,5]
	530.8	1.3	LiOH[3,5,6,7]
	531.9	1.3	Li_2CO_3[3,5,7,8]
	532.9	1.3	C-O[7,9,10,11]
	534.2	1.3	Unassigned
	536.2	1.6	Na Auger[12,13]
**N 1*s***	397.5	1.3	Li_3N[6,14,15,16]
	398.5	1.3	NH_2[17,18,19]
	399.6	1.3	NH_3[14,15]
	401	1.3	$Li_2N_2O_2$[20,21,22,23]
**C 1*s***	285	1.2	C-C[24,4,,25]
	286.8	1.2	C-O[25,24,26,27,28]
	288.3	1.2	C=O[24,4,26,27]
	289.4	1.2	CO_3[25,24,4,26,28]
**Li 1*s***	54.5	0.8	Li^0 [29,30,31,32]

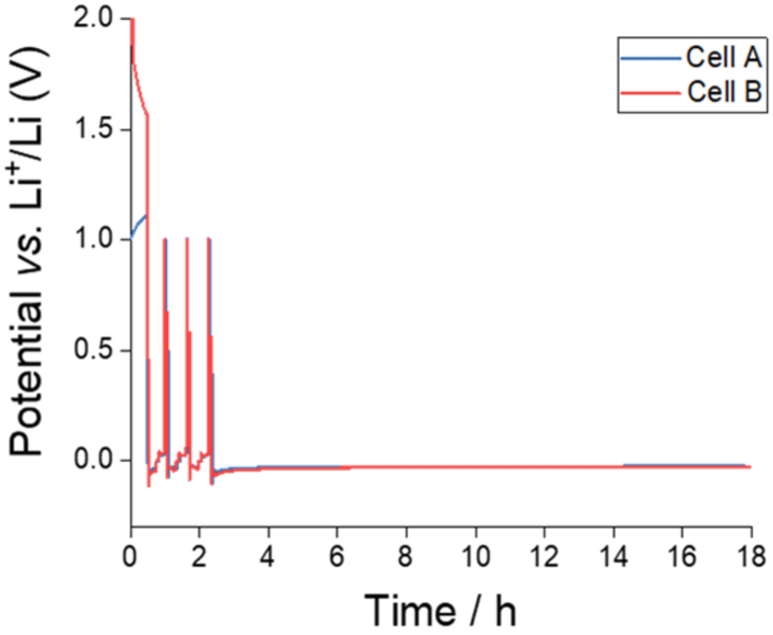

Figure S3. Voltage profiles of the electrodeposition process for two identical cells in forming plated lithium electrodes on Ni discs.

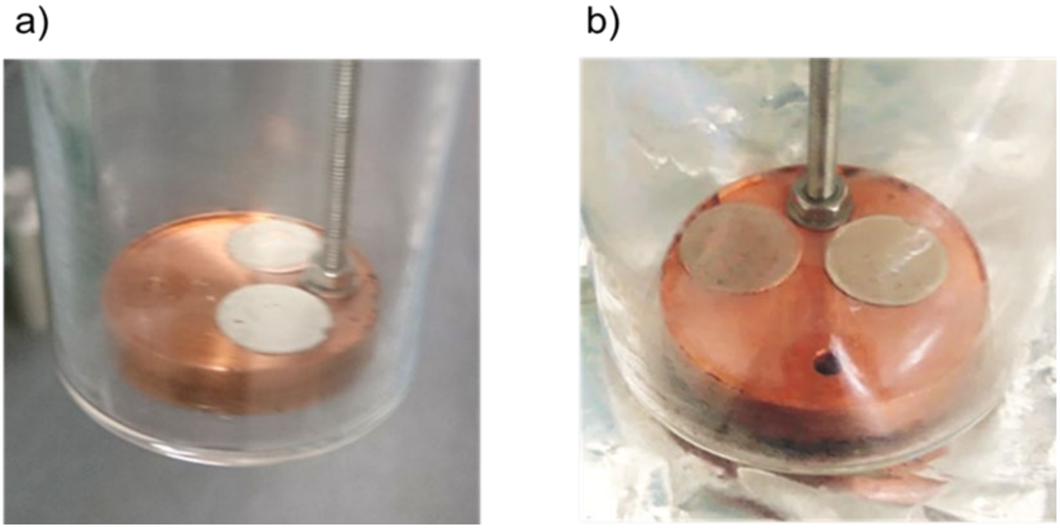

Figure S4. Electrodeposited lithium inside the portable reaction vessel before (**a**) and after (**b**) 2 hours nitridation at 90 °C.

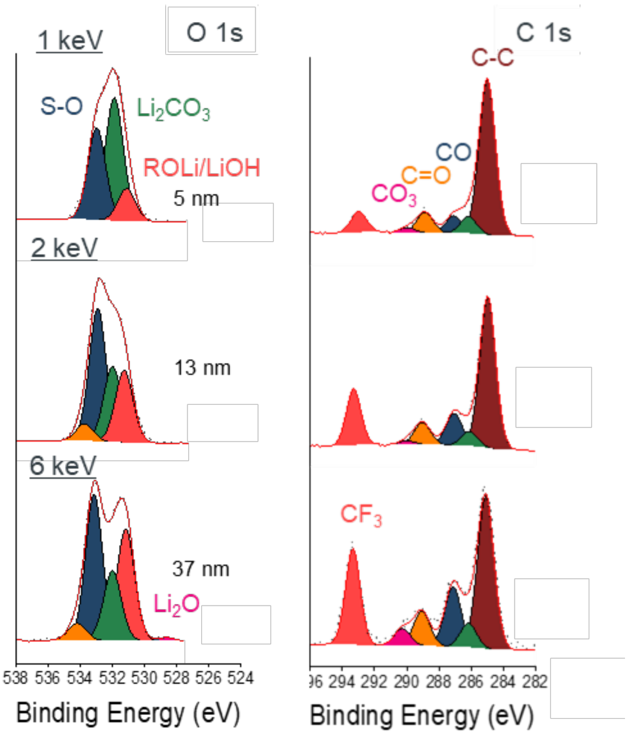

Figure S5. Synchrotron XPS spectra for the O 1*s*, and C 1*s* regions of the unmodified electrodeposited lithium electrode as a function of increasing excitation energies, with the calculated average probing depth provided for each spectrum.

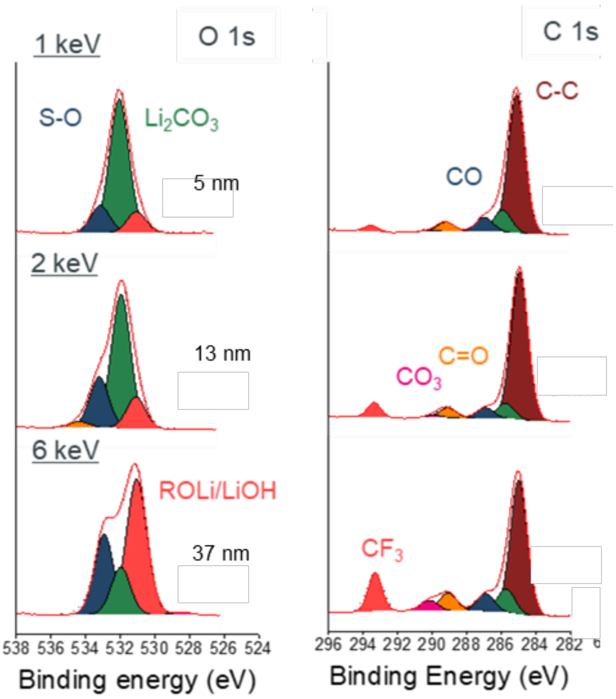

Figure S6. Synchrotron XPS spectra for the O 1*s* and C 1*s* regions of the nitrided electrodeposited lithium electrode as a function of increasing excitation energies, with the calculated average probing depth provided for each spectrum.

Table S4. Summary of XPS analysis and assignments for the surface characterisation of the unmodified and nitrided electrodeposited lithium electrode shown in Figures S5 and S6.

Orbital	Binding energy (eV)	FWHM (eV)	Peak assignments
**O 1*s***	528.3	1.2	Li_2O[3,4,5]
	530.8	1.3	ROLi/LiOH[3,6,7,33]
	531.9	1.3	Li_2CO_3[3,7,8]
	532.9	1.3	S-O / C-O[7,9,10,11,34]
	533.9	1.3	$LiTFSI_{decomp}$
**N 1*s***	397.5	1.3	Li_3N[6,14,15,16]
	398.8	1.3	$LiTFSI_{decomp}$[14]
	399.8	1.3	LiTFSI[6,14,35,36]
	401	1.3	$Li_2N_xO_y$[20,21,22,23]
**C 1*s***	285	1.2	C-C[24,4]
	286.8	1.2	C-O[25,24,27,28]
	288.3	1.2	C=O[24,4,27]
	289.4	1.2	CO_3[25,24,4,28]
	293.3	1.1	CF_3[37,26,38,39]
**Li 1*s***	54.5	0.8	Li^0 [29,30,31,32]

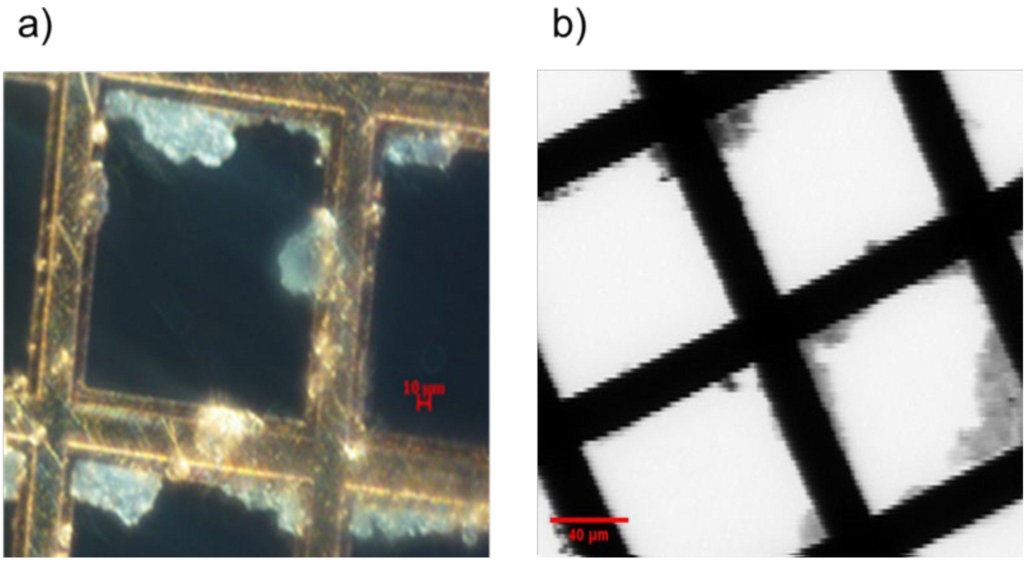

Figure S7. (**a**), Optical microscope image at 20x magnification of electrodeposited lithium on a nickel TEM grid. Scale bar = 10 µm. (**b**), 200 x 200 µm (pixel size = 500 nm) STXM image of electrodeposited lithium on a nickel TEM grid. Scale bar = 40 µm.

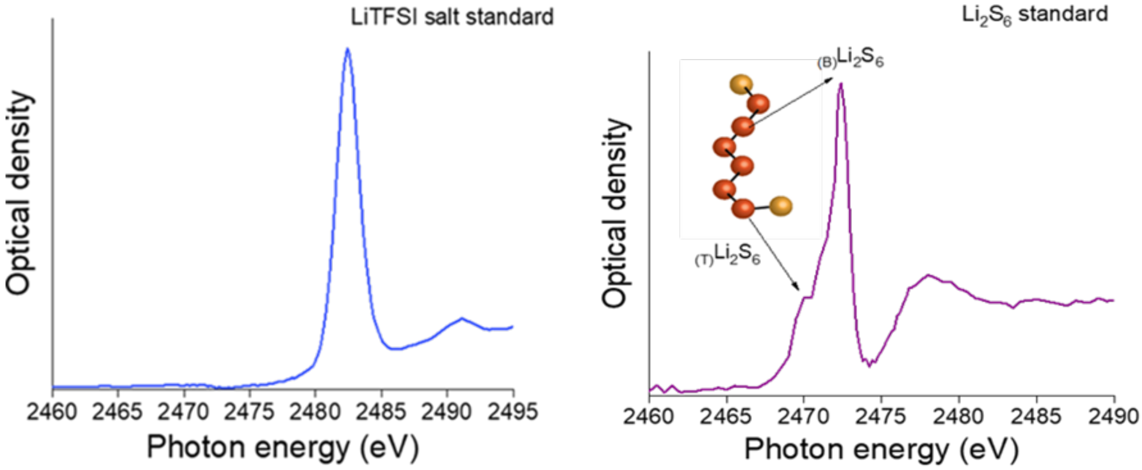

Figure S8. NEXAFS spectrum of the S K-edge energy for the (left) LiTFSI salt standard and (right) Li_2S_6 standard.

Table S5. Summary of XPS analysis and assignments for the surface characterisation of unmodified and nitrided electrodeposited lithium electrodes exposed to polysulfide solution.

Orbital	Binding energy (eV)	FWHM	Peak assignments
**<u>N 1*s*</u>**	397.5	1.3	Li_3N[6,14,15,16]
	398.8	1.3	$LiTFSI_{decomp}$[14]
	399.8	1.3	LiTFSI[6,14,26,35]
	401	1.3	Li_xNO_y[38,40,41]
**<u>S 2*p*</u>**	169.2	1.2	LiTFSI [34,42,43]
	167.2	1.2	Li_2SO_3[38,44,45,46]
	164	1.3	Metastable Li_2S_x (S_B) [47,34,42,43,48,49]
	162	1.3	Metastable Li_2S_x (S_T) [47,34,42,43,48,49]

Table S6. Summary of XPS analysis and assignments for the characterisation of unmodified and nitrided electrodeposited lithium electrodes exposed to polysulfide solution, and after 30 seconds of Ar^+ etching.

Orbital	Binding energy (eV)	FWHM	Peak assignments
**N 1*s***	397.5	1.3	Li_3N[6,14,15,16]
	398.8	1.3	$LiTFSI_{decomp}$[14]
	399.8	1.3	LiTFSI[6,14,26,35]
	401	1.3	Li_xNO_y[38,40,41]
**S 2*p***	169.2	1.1	LiTFSI [34,42,43]
	167.2	1.1	Li_2SO_3[38,44,45,46]
	166	1.1	Decomposition
	164	1	Metastable Li_2S_x (S_B) [47,34,42,43,48,49]
	162	1	Metastable Li_2S_x (S_T) [47,34,42,43,48,49]
	160.2	1	Li_2S[47,34,42,43,48,49]

Table S7. Summary of conventional XPS quantification of the unmodified and nitrided electrodeposited lithium electrodes exposed to polsulfide solution after 30 seconds to etching.

Orbital	Compounds	Atomic concentration (%) Unmodified	Atomic concentration (%) Nitrided
**N 1*s***	LiTFSI	10	7
	$LiTFSI_{decomposition}$	6	5
	Li_3N	3	4
	Li_2N_xOy	3	
**S 2*p***	LiTFSI	15	12
	$LiTFSI_{decomposition}$	9	9
	Decomposition product	3	5
	Li_2S_x (Bridging)	7	15
	Li_2S_x (Terminal)	17	34
	Li_2S	27	9

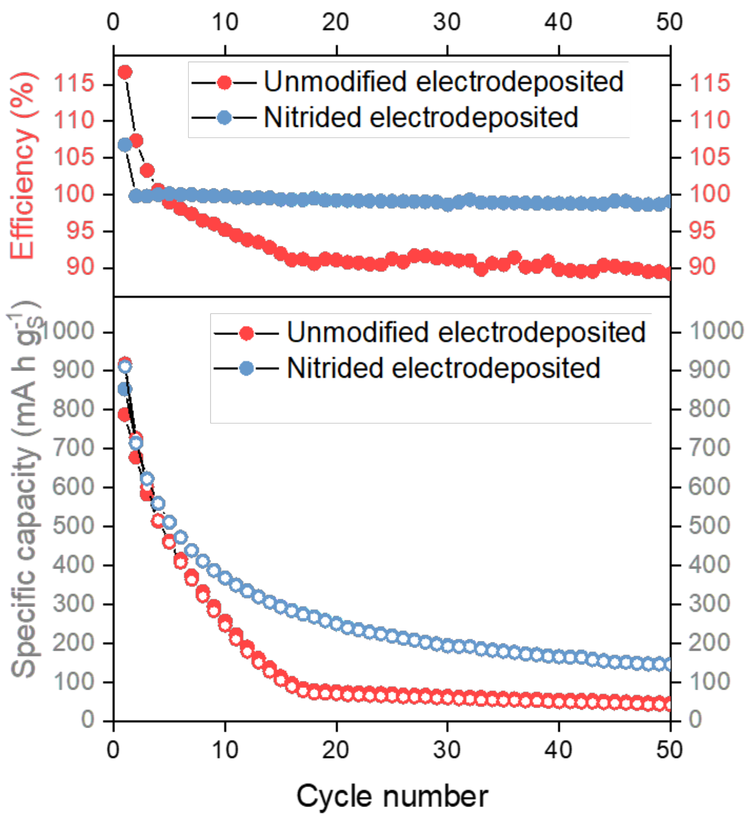

Figure S9. Cycling performance of Li-S cell assembled with an electrodeposited lithium anode that had been reacted with N_2 (nitride) or not (unmodified) at C/10.

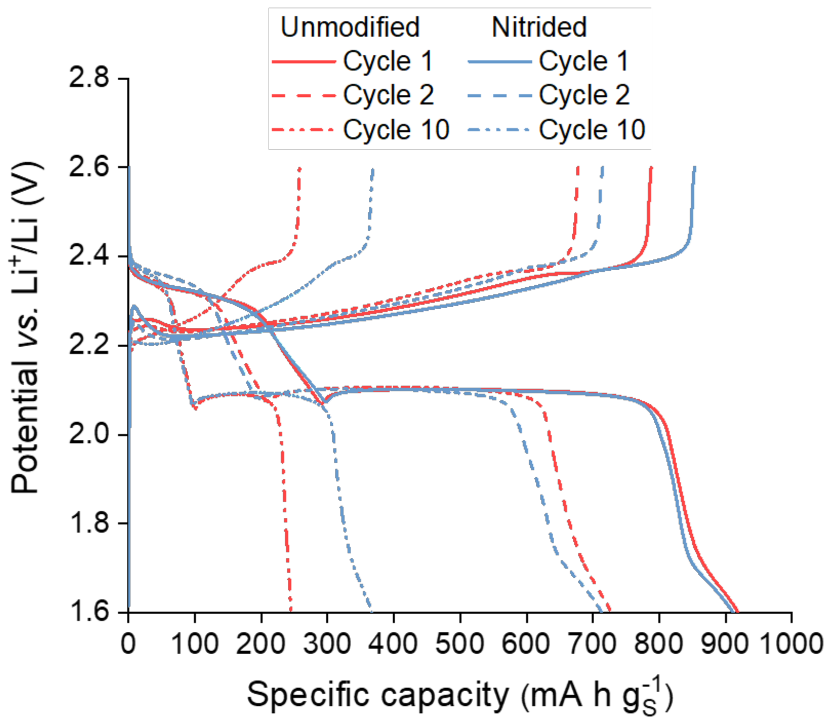

Figure S10. Voltage profile at selected cycles for the Li-S cells as C/10 in Figure S9.

References

(1) Tanuma, S.; Powell, C. J.; Penn, D. R. Calculations of Electron Inelastic Mean Free Paths. IX. Data for 41 Elemental Solids over the 50 EV to 30 KeV Range. *Surface and Interface Analysis* **2011**, *43* (3), 689–713. https://doi.org/10.1002/sia.3522.

(2) Jablonski, A.; Powell, C. J. Effective Attenuation Lengths for Different Quantitative Applications of X-Ray Photoelectron Spectroscopy. *Journal of Physical and Chemical Reference Data* **2020**, *49* (3), 033102. https://doi.org/10.1063/5.0008576.

(3) Becking, J.; Gröbmeyer, A.; Kolek, M.; Rodehorst, U.; Schulze, S.; Winter, M.; Bieker, P.; Stan, M. C. Lithium-Metal Foil Surface Modification: An Effective Method to Improve the Cycling Performance of Lithium-Metal Batteries. *Advanced Materials Interfaces* **2017**, *4* (16), 1700166. https://doi.org/10.1002/admi.201700166.

(4) Xiong, S.; Xie, K.; Diao, Y.; Hong, X. Characterization of the Solid Electrolyte Interphase on Lithium Anode for Preventing the Shuttle Mechanism in Lithium–Sulfur Batteries. *Journal of Power Sources* **2014**, *246*, 840–845. https://doi.org/10.1016/j.jpowsour.2013.08.041.

(5) Ismail, I.; Noda, A.; Nishimoto, A.; Watanabe, M. XPS Study of Lithium Surface after Contact with Lithium-Salt Doped Polymer Electrolytes. *Electrochimica Acta* **2001**, *46* (10), 1595–1603. https://doi.org/10.1016/S0013-4686(00)00758-1.

(6) Xu, C.; Sun, B.; Gustafsson, T.; Edström, K.; Brandell, D.; Hahlin, M. Interface Layer Formation in Solid Polymer Electrolyte Lithium Batteries: An XPS Study. *J. Mater. Chem. A* **2014**, *2* (20), 7256–7264. https://doi.org/10.1039/C4TA00214H.

(7) Malmgren, S.; Ciosek, K.; Lindblad, R.; Plogmaker, S.; Kühn, J.; Rensmo, H.; Edström, K.; Hahlin, M. Consequences of Air Exposure on the Lithiated Graphite SEI. *Electrochimica Acta* **2013**, *105*, 83–91. https://doi.org/10.1016/j.electacta.2013.04.118.

(8) Deng, B.; Wang, H.; Ge, W.; Li, X.; Yan, X.; Chen, T.; Qu, M.; Peng, G. Investigating the Influence of High Temperatures on the Cycling Stability of a LiNi0.6Co0.2Mn0.2O2 Cathode Using an Innovative Electrolyte Additive. *Electrochimica Acta* **2017**, *236*, 61–71. https://doi.org/10.1016/j.electacta.2017.03.155.

(9) Wang, X.; Sun, Z.; Zhao, Y.; Li, J.; Zhang, Y.; Zhang, Z. Na4Mn9O18 Nanowires Wrapped by Reduced Graphene Oxide as Efficient Sulfur Host Material for Lithium/Sulfur Batteries. *J Solid State Electrochem* **2020**, *24* (1), 111–119. https://doi.org/10.1007/s10008-019-04478-0.

(10) Farhat, D.; Ghamouss, F.; Maibach, J.; Edström, K.; Lemordant, D. Adiponitrile–Lithium Bis(Trimethylsulfonyl)Imide Solutions as Alkyl Carbonate-Free Electrolytes for Li4Ti5O12 (LTO)/LiNi1/3Co1/3Mn1/3O2 (NMC) Li-Ion Batteries. *ChemPhysChem* **2017**, *18* (10), 1333–1344. https://doi.org/10.1002/cphc.201700058.

(11) Li, X.; Yin, Z.; Li, X.; Wang, C. Ethylene Sulfate as Film Formation Additive to Improve the Compatibility of Graphite Electrode for Lithium-Ion Battery. *Ionics* **2014**, *20* (6), 795–801. https://doi.org/10.1007/s11581-013-1036-5.

(12) Miura, Y.; Kusano, H.; Nanba, T.; Matsumoto, S. X-Ray Photoelectron Spectroscopy of Sodium Borosilicate Glasses. *Journal of Non-Crystalline Solids* **2001**, *290* (1), 1–14. https://doi.org/10.1016/S0022-3093(01)00720-7.

(13) Baggetto, L.; Ganesh, P.; Sun, C.-N.; A. Meisner, R.; A. Zawodzinski, T.; M. Veith, G. Intrinsic Thermodynamic and Kinetic Properties of Sb Electrodes for Li-Ion and Na-Ion Batteries: Experiment and Theory. *Journal of Materials Chemistry A* **2013**, *1* (27), 7985–7994. https://doi.org/10.1039/C3TA11568B.

(14) Grissa, R.; Fernandez, V.; Fairley, N.; Hamon, J.; Stephant, N.; Rolland, J.; Bouchet, R.; Lecuyer, M.; Deschamps, M.; Guyomard, D.; Moreau, P. XPS and SEM-EDX Study of

Electrolyte Nature Effect on Li Electrode in Lithium Metal Batteries. *ACS Appl. Energy Mater.* **2018**, *1* (10), 5694–5702. https://doi.org/10.1021/acsaem.8b01256.
(15) Zhao, C.-Z.; Cheng, X.-B.; Zhang, R.; Peng, H.-J.; Huang, J.-Q.; Ran, R.; Huang, Z.-H.; Wei, F.; Zhang, Q. Li2S5-Based Ternary-Salt Electrolyte for Robust Lithium Metal Anode. *Energy Storage Materials* **2016**, *3*, 77–84. https://doi.org/10.1016/j.ensm.2016.01.007.
(16) Xue, C.; Zhang, X.; Wang, S.; Li, L.; Nan, C.-W. Organic–Organic Composite Electrolyte Enables Ultralong Cycle Life in Solid-State Lithium Metal Batteries. *ACS Appl. Mater. Interfaces* **2020**, *12* (22), 24837–24844. https://doi.org/10.1021/acsami.0c05643.
(17) Al-Ani, A.; Pingle, H.; P Reynolds, N.; Wang, P.-Y.; Kingshott, P. Tuning the Density of Poly(Ethylene Glycol) Chains to Control Mammalian Cell and Bacterial Attachment. *Polymers* **2017**, *9* (8), 343. https://doi.org/10.3390/polym9080343.
(18) Ravi, S.; Zhang, S.; Lee, Y.-R.; Kang, K.-K.; Kim, J.-M.; Ahn, J.-W.; Ahn, W.-S. EDTA-Functionalized KCC-1 and KIT-6 Mesoporous Silicas for Nd3+ Ion Recovery from Aqueous Solutions. *Journal of Industrial and Engineering Chemistry* **2018**, *67*, 210–218. https://doi.org/10.1016/j.jiec.2018.06.031.
(19) Xie, Y.; Fang, L.; Cheng, H.; Hu, C.; Zhao, H.; Xu, J.; Fang, J.; Lu, X.; Zhang, J. Biological Cell Derived N-Doped Hollow Porous Carbon Microspheres for Lithium–Sulfur Batteries. *J. Mater. Chem. A* **2016**, *4* (40), 15612–15620. https://doi.org/10.1039/C6TA06164H.
(20) Zhang, A.; Fang, X.; Shen, C.; Liu, Y.; Zhou, C. A Carbon Nanofiber Network for Stable Lithium Metal Anodes with High Coulombic Efficiency and Long Cycle Life. *Nano Res.* **2016**, *9* (11), 3428–3436. https://doi.org/10.1007/s12274-016-1219-2.
(21) Chen, X.; Hou, T.-Z.; Li, B.; Yan, C.; Zhu, L.; Guan, C.; Cheng, X.-B.; Peng, H.-J.; Huang, J.-Q.; Zhang, Q. Towards Stable Lithium-Sulfur Batteries: Mechanistic Insights into Electrolyte Decomposition on Lithium Metal Anode. *Energy Storage Materials* **2017**, *8*, 194–201. https://doi.org/10.1016/j.ensm.2017.01.003.
(22) Xing, X.; Li, Y.; Wang, X.; Petrova, V.; Liu, H.; Liu, P. Cathode Electrolyte Interface Enabling Stable Li–S Batteries. *Energy Storage Materials* **2019**, *21*, 474–480. https://doi.org/10.1016/j.ensm.2019.06.022.
(23) Liu, S.; Xia, X.; Deng, S.; Xie, D.; Yao, Z.; Zhang, L.; Zhang, S.; Wang, X.; Tu, J. In Situ Solid Electrolyte Interphase from Spray Quenching on Molten Li: A New Way to Construct High-Performance Lithium-Metal Anodes. *Advanced Materials* **2019**, *31* (3), 1806470. https://doi.org/10.1002/adma.201806470.
(24) Fang, C.; Li, J.; Zhang, M.; Zhang, Y.; Yang, F.; Lee, J. Z.; Lee, M.-H.; Alvarado, J.; Schroeder, M. A.; Yang, Y.; Lu, B.; Williams, N.; Ceja, M.; Yang, L.; Cai, M.; Gu, J.; Xu, K.; Wang, X.; Meng, Y. S. Quantifying Inactive Lithium in Lithium Metal Batteries. *Nature* **2019**, *572* (7770), 511–515. https://doi.org/10.1038/s41586-019-1481-z.
(25) Xiong, S.; Xie, K.; Diao, Y.; Hong, X. Properties of Surface Film on Lithium Anode with LiNO3 as Lithium Salt in Electrolyte Solution for Lithium–Sulfur Batteries. *Electrochimica Acta* **2012**, *83*, 78–86. https://doi.org/10.1016/j.electacta.2012.07.118.
(26) Fiedler, C.; Luerssen, B.; Rohnke, M.; Sann, J.; Janek, J. XPS and SIMS Analysis of Solid Electrolyte Interphases on Lithium Formed by Ether-Based Electrolytes. *J. Electrochem. Soc.* **2017**, *164* (14), A3742–A3749. https://doi.org/10.1149/2.0851714jes.
(27) Cheng, X.-B.; Yan, C.; Chen, X.; Guan, C.; Huang, J.-Q.; Peng, H.-J.; Zhang, R.; Yang, S.-T.; Zhang, Q. Implantable Solid Electrolyte Interphase in Lithium-Metal Batteries. *Chem* **2017**, *2* (2), 258–270. https://doi.org/10.1016/j.chempr.2017.01.003.

(28) Xiong, S.; Xie, K.; Diao, Y.; Hong, X. On the Role of Polysulfides for a Stable Solid Electrolyte Interphase on the Lithium Anode Cycled in Lithium–Sulfur Batteries. *Journal of Power Sources* **2013**, *236*, 181–187. https://doi.org/10.1016/j.jpowsour.2013.02.072.
(29) Wood, K. N.; Steirer, K. X.; Hafner, S. E.; Ban, C.; Santhanagopalan, S.; Lee, S.-H.; Teeter, G. Operando X-Ray Photoelectron Spectroscopy of Solid Electrolyte Interphase Formation and Evolution in Li 2 S-P 2 S 5 Solid-State Electrolytes. *Nature Communications* **2018**, *9* (1), 1–10. https://doi.org/10.1038/s41467-018-04762-z.
(30) Mallinson, C. F.; Castle, J. E.; Watts, J. F. Analysis of the Li KLL Auger Transition on Freshly Exposed Lithium and Lithium Surface Oxide by AES. *Surface Science Spectra* **2013**, *20* (1), 113–127. https://doi.org/10.1116/11.20130901.
(31) Zhang, Y.; Xia, X.; Wang, D.; Wang, X.; Gu, C.; Tu, J. Integrated Reduced Graphene Oxide Multilayer/Li Composite Anode for Rechargeable Lithium Metal Batteries. *RSC Adv.* **2016**, *6* (14), 11657–11664. https://doi.org/10.1039/C5RA25553H.
(32) Oswald, S.; Thoss, F.; Zier, M.; Hoffmann, M.; Jaumann, T.; Herklotz, M.; Nikolowski, K.; Scheiba, F.; Kohl, M.; Giebeler, L.; Mikhailova, D.; Ehrenberg, H. Binding Energy Referencing for XPS in Alkali Metal-Based Battery Materials Research (II): Application to Complex Composite Electrodes. *Batteries* **2018**, *4* (3), 36. https://doi.org/10.3390/batteries4030036.
(33) Jiao, S.; Ren, X.; Cao, R.; Engelhard, M. H.; Liu, Y.; Hu, D.; Mei, D.; Zheng, J.; Zhao, W.; Li, Q.; Liu, N.; Adams, B. D.; Ma, C.; Liu, J.; Zhang, J.-G.; Xu, W. Stable Cycling of High-Voltage Lithium Metal Batteries in Ether Electrolytes. *Nature Energy* **2018**, *3* (9), 739–746. https://doi.org/10.1038/s41560-018-0199-8.
(34) Nandasiri, M. I.; Camacho-Forero, L. E.; Schwarz, A. M.; Shutthanandan, V.; Thevuthasan, S.; Balbuena, P. B.; Mueller, K. T.; Murugesan, V. In Situ Chemical Imaging of Solid-Electrolyte Interphase Layer Evolution in Li–S Batteries. *Chem. Mater.* **2017**, *29* (11), 4728–4737. https://doi.org/10.1021/acs.chemmater.7b00374.
(35) Dedryvère, R.; Leroy, S.; Martinez, H.; Blanchard, F.; Lemordant, D.; Gonbeau, D. XPS Valence Characterization of Lithium Salts as a Tool to Study Electrode/Electrolyte Interfaces of Li-Ion Batteries. *J. Phys. Chem. B* **2006**, *110* (26), 12986–12992. https://doi.org/10.1021/jp061624f.
(36) Busche, M. R.; Drossel, T.; Leichtweiss, T.; Weber, D. A.; Falk, M.; Schneider, M.; Reich, M.-L.; Sommer, H.; Adelhelm, P.; Janek, J. Dynamic Formation of a Solid-Liquid Electrolyte Interphase and Its Consequences for Hybrid-Battery Concepts. *Nature Chemistry* **2016**, *8* (5), 426–434. https://doi.org/10.1038/nchem.2470.
(37) Aurbach, D. Review of Selected Electrode–Solution Interactions Which Determine the Performance of Li and Li Ion Batteries. *Journal of Power Sources* **2000**, *89* (2), 206–218. https://doi.org/10.1016/S0378-7753(00)00431-6.
(38) Aurbach, D.; Pollak, E.; Elazari, R.; Salitra, G.; Kelley, C. S.; Affinito, J. On the Surface Chemical Aspects of Very High Energy Density, Rechargeable Li–Sulfur Batteries. *J. Electrochem. Soc.* **2009**, *156* (8), A694. https://doi.org/10.1149/1.3148721.
(39) Nilsson, V.; Younesi, R.; Brandell, D.; Edström, K.; Johansson, P. Critical Evaluation of the Stability of Highly Concentrated LiTFSI - Acetonitrile Electrolytes vs. Graphite, Lithium Metal and LiFePO4 Electrodes. *Journal of Power Sources* **2018**, *384*, 334–341. https://doi.org/10.1016/j.jpowsour.2018.03.019.
(40) Jie, Y.; Xu, Y.; Chen, Y.; Xie, M.; Liu, Y.; Huang, F.; Kochovski, Z.; Lei, Z.; Zheng, L.; Song, P.; Hu, C.; Qi, Z.; Li, X.; Wang, S.; Shen, Y.; Chen, L.; You, Y.; Ren, X.; Goddard, W. A.; Cao, R.; Lu, Y.; Cheng, T.; Xu, K.; Jiao, S. Molecular Understanding of Interphase Formation via Operando Polymerization on Lithium Metal Anode. *Cell*

Reports Physical Science **2022**, *3* (10), 101057. https://doi.org/10.1016/j.xcrp.2022.101057.
(41) Wen, Y.; Ding, J.; Liu, J.; Zhu, M.; Hu, R. A Separator Rich in SnF2 and NO3− Directs an Ultra-Stable Interface toward High Performance Li Metal Batteries. *Energy Environ. Sci.* **2023**. https://doi.org/10.1039/D3EE00664F.
(42) Zu, C.; Azimi, N.; Zhang, Z.; Manthiram, A. Insight into Lithium–Metal Anodes in Lithium–Sulfur Batteries with a Fluorinated Ether Electrolyte. *Journal of Materials Chemistry A* **2015**, *3* (28), 14864–14870. https://doi.org/10.1039/C5TA03195H.
(43) Liang, X.; Kwok, C. Y.; Lodi-Marzano, F.; Pang, Q.; Cuisinier, M.; Huang, H.; Hart, C. J.; Houtarde, D.; Kaup, K.; Sommer, H.; Brezesinski, T.; Janek, J.; Nazar, L. F. Tuning Transition Metal Oxide–Sulfur Interactions for Long Life Lithium Sulfur Batteries: The "Goldilocks" Principle. *Advanced Energy Materials* **2016**, *6* (6), 1501636. https://doi.org/10.1002/aenm.201501636.
(44) Lacey, M. J.; Yalamanchili, A.; Maibach, J.; Tengstedt, C.; Edström, K.; Brandell, D. The Li–S Battery: An Investigation of Redox Shuttle and Self-Discharge Behaviour with LiNO3-Containing Electrolytes. *RSC Adv.* **2016**, *6* (5), 3632–3641. https://doi.org/10.1039/C5RA23635E.
(45) Chien, Y.-C.; Pan, R.; Lee, M.-T.; Nyholm, L.; Brandell, D.; Lacey, M. J. Cellulose Separators With Integrated Carbon Nanotube Interlayers for Lithium-Sulfur Batteries: An Investigation into the Complex Interplay between Cell Components. *J. Electrochem. Soc.* **2019**, *166* (14), A3235. https://doi.org/10.1149/2.0301914jes.
(46) Lee, M.-T.; Liu, H.; Brandell, D. The Surface Chemistry of Thin Lithium Metal Electrodes in Lithium-Sulfur Cells. *Batteries & Supercaps* **2020**, *3* (12), 1370–1376. https://doi.org/10.1002/batt.202000145.
(47) Liang, X.; Hart, C.; Pang, Q.; Garsuch, A.; Weiss, T.; Nazar, L. F. A Highly Efficient Polysulfide Mediator for Lithium–Sulfur Batteries. *Nature Communications* **2015**, *6* (1), 1–8. https://doi.org/10.1038/ncomms6682.
(48) Klein, M. J.; Goossens, K.; Bielawski, C. W.; Manthiram, A. Elucidating the Electrochemical Activity of Electrolyte-Insoluble Polysulfide Species in Lithium-Sulfur Batteries. *J. Electrochem. Soc.* **2016**, *163* (9), A2109. https://doi.org/10.1149/2.0051610jes.
(49) Fantauzzi, M.; Elsener, B.; Atzei, D.; Rigoldi, A.; Rossi, A. Exploiting XPS for the Identification of Sulfides and Polysulfides. *RSC Adv.* **2015**, *5* (93), 75953–75963. https://doi.org/10.1039/C5RA14915K.
